# Cooperation of Angiopoietin-2 and Angiopoietin-4 in Schlemm's Canal Maintenance

**DOI:** 10.1167/iovs.63.11.1

**Published:** 2022-10-03

**Authors:** Emmi Kapiainen, Harri Elamaa, Ilkka Miinalainen, Valerio Izzi, Lauri Eklund

**Affiliations:** 1 Oulu Center for Cell-Matrix Research, University of Oulu, Oulu, Finland; 2 Faculty of Biochemistry and Molecular Medicine, University of Oulu, Oulu, Finland; 3 Biocenter Oulu, University of Oulu, Oulu, Finland; 4 Faculty of Medicine, University of Oulu, Oulu, Finland; 5 Foundation for the Finnish Cancer Institute, Helsinki, Finland

**Keywords:** Schlemm's canal, Tie2, angiopoietin, trabecular meshwork, glaucoma

## Abstract

**Purpose:**

Defects in the iridocorneal angle tissues, including the trabecular meshwork (TM) and Schlemm's canal (SC), impair aqueous humor flow and increase the intraocular pressure (IOP), eventually resulting in glaucoma. Activation of endothelial tyrosine kinase receptor Tie2 by angiopoietin-1 (Angpt1) has been demonstrated to be essential for SC formation, but roles of the other two Tie2 ligands, Angpt2 and Angpt4, have been controversial or not yet characterized, respectively.

**Methods:**

Angpt4 expression was investigated using genetic cell fate mapping and reporter mice. Congenital deletion of *Angpt2* and *Angpt4* and tamoxifen-inducible deletion of *Angpt1* in mice were used to study the effects of *Angpt4* deletion alone and in combination with the other angiopoietins. SC morphology was examined with immunofluorescent staining. IOP measurements, electron microscopy, and histologic evaluation were used to study glaucomatous changes.

**Results:**

*Angpt4* was postnatally expressed in the TM. While *Angpt4* deletion alone did not affect SC and *Angpt4* deletion did not aggravate *Angpt1* deletion phenotype, absence of *Angpt4* combined with *Angpt2* deletion had detrimental effects on SC morphology in adult mice. Consequently, *Angpt2*^−/−^;*Angpt4*^−/−^ mice displayed glaucomatous changes in the eye. Mice with *Angpt2* deletion alone showed only moderate SC defects, but Angpt2 was necessary for proper limbal vasculature development. Mechanistically, analysis of Tie2 phosphorylation suggested that Angpt2 and Angpt4 cooperate as agonistic Tie2 ligands in maintaining SC integrity.

**Conclusions:**

Our results indicated an additive effect of Angpt4 in SC maintenance and Tie2 activation and a spatiotemporally regulated interplay between the angiopoietins in the mouse iridocorneal angle.

Balanced outflow of aqueous humor (AH) in the eye is critical for normal vision. Impaired AH outflow increases the intraocular pressure (IOP), which is a significant risk factor for glaucoma, a heterogenous group of ocular diseases that are the major cause of irreversible blindness worldwide.^1^^–^^3^ Genetic studies have revealed genes essential for the proper development of the iridocorneal angle tissues and mutations causing increased resistance and reduced AH outflow.^2^^,^^4^^–^^9^ However, not all affecting genetic and physiologic factors are currently known.^3^ Thus, full characterization of the normal development and maintenance of the AH drainage system and the pathophysiologic mechanisms resulting in its increased resistance are needed to understand how AH drainage occurs, which should also help in developing new pharmacologic treatments to alleviate the increase of IOP.

The main exit route for AH, produced by the ciliary processes, drains from the anterior chamber via the trabecular meshwork (TM) to Schlemm's canal (SC) and from there to the aqueous and episcleral veins.^10^^,^^11^ AH is filtered to the SC through TM, which carefully adjusts the resistance for AH outflow.^11^^,^^12^ SC is a highly specialized vessel encircling the corneal limbus area and expressing a unique combination of both lymphatic vessel (LV) and blood vessel (BV) markers.^4^^,^^9^^,^^13^^–^^17^ Functionally, SC more resembles LVs and maintains optimal AH flow.^13^^–^^15^

The vascular angiopoietin (Angpt)/Tie signaling pathway consists of three ligands, Angpt1, Angpt2, and Angpt4; the primary Angpt receptor tyrosine kinase Tie2; and an orphan regulatory receptor Tie1.^18^ While Angpt1 is the main agonistic Tie2 ligand mediating endothelial cell survival and stability, Angpt2 is a context-dependent Tie2 agonist/antagonist involved in vascular remodeling.^18^ Angpt2 is generally considered to inhibit Tie2 activation by Angpt1 in the BVs but to be capable of inducing Tie2 activation in the LVs.^19^^–^^22^ Angpt4 was recently identified to mediate venous development in the mouse retina,^23^ but its role in fluid drainage in other tissues is not reported.

Compelling evidence from recent years shows that Tie2 activation is essential for both SC development and its maintenance. *Tie2* deletion as well as *Angpt1;Angpt2* double deletion in mice lead to complete absence of SC, highly elevated IOP, and severe glaucoma.^16^^,^^24^^–^^26^ *Tie2* heterozygosity or *Angpt1* deletion is sufficient to partially degenerate SC in mice,^16^^,^^17^^,^^25^^–^^27^ indicating dose dependency of required Tie2 activation. This is also true in patients heterozygous for *TIE2* and *ANGPT1* loss-of-function mutations who have been diagnosed with glaucoma.^16^^,^^25^^,^^28^ In addition, *TIE2* and *ANGPT1* variants have been associated with increased IOP and glaucoma in genome-wide association studies (GWASs).^6^^–^^8^ Along with this, inhibition of Tie2-dephosphorylating vascular endothelial protein tyrosine phosphatase VE-PTP has been shown to be a potential treatment to increase Tie2 activation and partly protect from glaucomatous changes.^29^^,^^30^ Very recently, also Tie1, which can potentiate Tie2 activation, was described as a necessary factor for SC development.^31^

However, among the Angpt ligands, the importance of Angpt4 in the iridocorneal angle has not been investigated, and the individual role of Angpt2 has remained somewhat controversial. Kim et al.^26^ reported decreased SC and corneal limbus LV area in *Angpt2* deletion mice, and *ANGPT2* variant loci have been associated with increased IOP in glaucoma patient GWAS analyses.^5^^–^^8^ In contrast, Thomson et al.^16^ found no difference in either IOP or SC area in *Angpt2* deletion mice, and administration of Angpt2-blocking antibody has been reported to have no effect on IOP in monkeys or on SC area in mice.^32^

Here, we used genetic mouse models to study the importance of Angpt4 in AH drainage. We show that Angpt4 expression is spatiotemporally distinct form the other Angpts in the iridocorneal angle, and while *Angpt4* deletion alone did not affect the SC, its deletion combined with *Angpt2* deletion had detrimental effects on both SC morphology and AH outflow, causing glaucomatous changes in the eye. Furthermore, being the first to use congenital *Angpt2* deletion in this context, we show that Angpt2 is indeed necessary for normal SC morphology and also for the corneolimbal vasculature. Collectively, our data establish a novel role for Angpt4 in the iridocorneal angle and reveal cooperation between Angpt2 and Angpt4 necessary for full Tie2 activation and SC maintenance.

## Materials and Methods

### Generation and Maintenance of Mouse Lines

All animal experiments were performed with the approval of the Finnish Project Authorization Board following national and European Union (EU)–level legislation (EU directive 2010/63/EU) and adhering to the ARVO Statement for the Use of Animals in Ophthalmic and Vision Research. *Angpt2* deletion mice were generated by CRISPR/Cas9-based genome editing.^33^^–^^36^ The CRISPR guideRNA was designed with a guideRNA tool (Crispr.mit.edu, ACTGAGTCGTCGTAGTCGAG). Based on that analysis, CRISPR RNA (crRNA), trans-activating CRISPR RNA (tracrRNA), and Cas9 protein (Cas9 nuclease) were ordered from Integrated DNA Technologies (Coralville, IA, USA). Ribonucleoprotein complex (20 ng/µL crRNA/tracrRNA), together with Cas9 protein (20 ng/µL), was injected into C3H/HeNCrl (Charles River Laboratories, Wilmington, MA, USA) mouse zygotes. Microinjections were performed at the Biocenter Oulu Transgenic and Tissue Phenotyping Core Facility, University of Oulu, Finland. Embryos were transferred to pseudo-pregnant CD1 females, and founders were screened by sequencing and T7 endonuclease I digestion of the PCR product from the modified region. A 35-bp deletion was confirmed at the cDNA level from the heart, lung, and kidney samples by sequencing. Founders were mated with wild-type (WT) C57BL/6NCrl mice (Charles River Laboratories) for at least six breeding rounds to remove off-targets in the genomic DNA.

Floxed *Angpt1* allele (referred as *Angpt1*^fl^ throughout the text) was ordered from The Jackson Laboratory (Bar Harbor, ME, USA) under a strain name *Angpt1*^tm2.1Sjm^/J (#028925).^37^ *Rosa26*^CreERT2^ mice expressing tamoxifen-inducible Cre under ubiquitous *Rosa26* promoter were kindly provided by the GIE-CERBM (GIE-Centre Européen de Recherche en Biologie et en Médecine, Strasbourg, France). Generation of the *Angpt4* knockout allele (*Angpt4*^Cre/Cre^, referred as *Angpt4*^−/−^ throughout the text) has been described previously.^23^ These lines were crossed to obtain the *Angpt1*^fl/fl^*;Angpt4*^−^*^/^*^−^*;Rosa26*^CreERT2^ mouse line, and the mice were administered tamoxifen (4 mg; T5648; Sigma Aldrich, St. Louis, MO, USA) by oral gavaging starting from 4 weeks of age and continued at a 1-week interval until 8 weeks of age to delete *Angpt1* and create an *Angpt1*^del^*;Angpt4*^−^*^/^*^−^ mouse line*.* Littermates received the same tamoxifen administrations and were used as controls. *Angpt4*^Cre/+^*;Rosa26*^mTmG/+^ cell fate mapping and *Angpt4*^LacZ^ reporter mice have been introduced before.^23^^,^^38^

Primers used for genotyping the herein introduced mouse lines are presented in the Supplementary Table S1. All mice were kept in the C57BL/6NCrl background and given unrestricted access to standard rodent chow and water. Both male and female mice were used as it has been shown in a large follow-up study that IOP values do not differ between males and females in the C57BL/6N background,^39^ being in accordance with our own observations and the practice of other similar studies.^16^^,^^17^^,^^27^

### Histologic Stainings, Imaging, and Tissue Analyses

Detailed protocols for immunofluorescence whole-mount and section stainings, X-gal staining, hematoxylin and eosin staining, confocal microscopy, transmission electron microscopy, all image analyses, single-cell RNA sequencing (scRNAseq) data analysis, and quantitative PCR (qPCR) are given in the Supplementary Materials and Methods. One eye/mouse was used in each histologic analysis and quantitated data set.

### IOP Measurements

IOP was measured using a Tonolab rebound tonometer (iCare, Vantaa, Finland). Mice were accustomed to handling prior to the measurements. While measuring (consistently around noon), mice were restrained by an experienced animal caretaker, and IOP values were obtained from two to three sets of six recordings, which were finally averaged from both eyes to obtain a single value for each mouse.^16^^,^^17^^,^^27^^,^^29^^,^^39^

### Statistics

Normal distribution of the data was confirmed with the Shapiro–Wilk test for normality performed in Prism 9 software (GraphPad Software, San Diego, CA, USA), and equal variance was tested with Levene's test for homogeneity of variance using Origin Pro software (OriginLab, Northampton, MA, USA). When both assumptions were met, comparisons between multiple groups were performed with one-way ANOVA followed by the Tukey post hoc test, and when variances were unequal (indicated in the figure legends), Welch's ANOVA followed by Dunnett's T3 multiple comparisons test was performed using Prism 9 software. Unpaired two-tailed Student's *t*-test was used for comparing two groups after confirming normal distribution and equal variance. Statistical significances are marked in figures as following: *P < 0.05, **P < 0.01, and ***P < 0.001, and all figures represent individual data points and mean ± SD.

## Results

### Angiopoietin-4 is Postnatally Expressed in the Mouse Iridocorneal Angle

**Figure 1.**
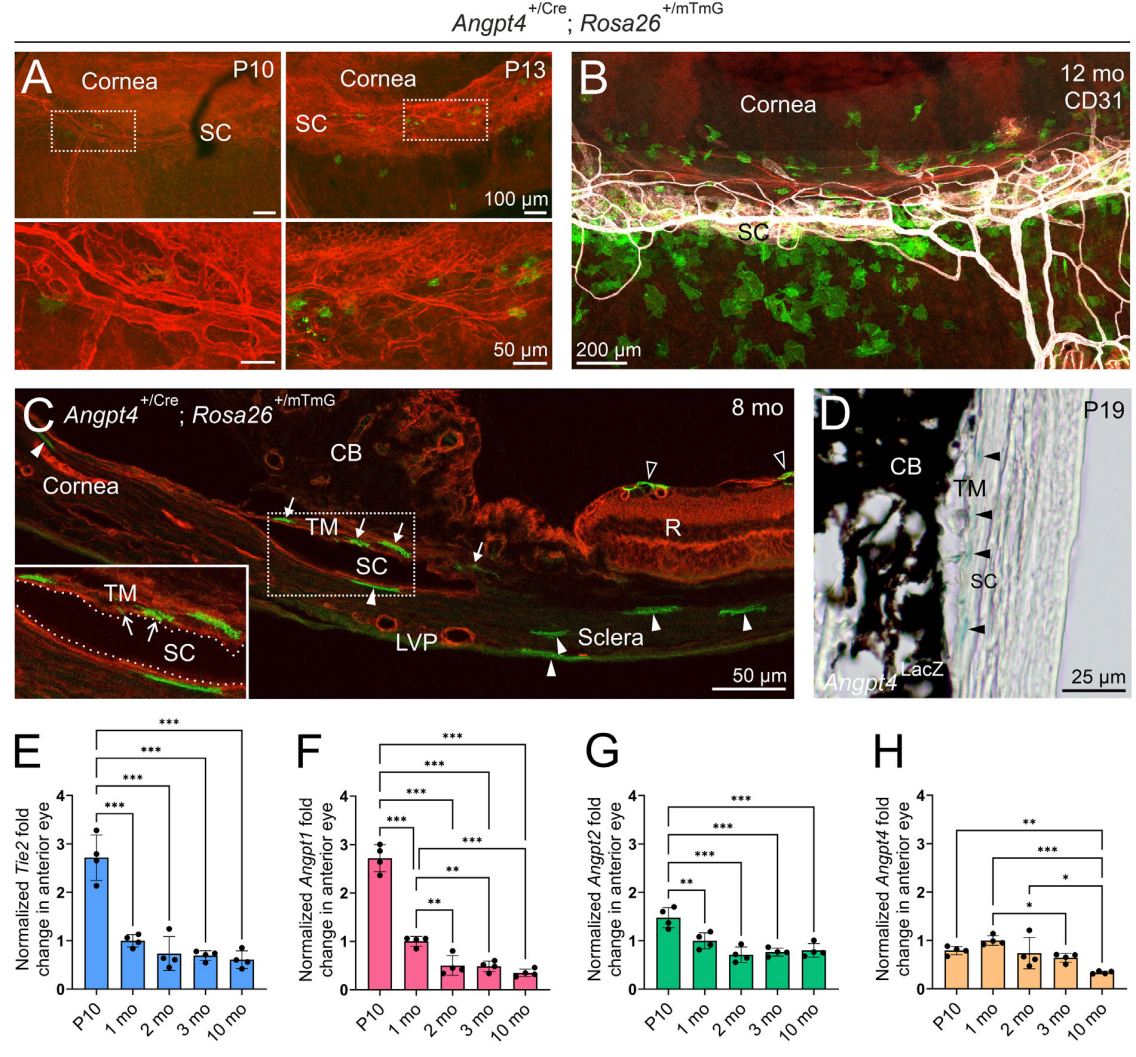
*Angpt4* expression in the mouse iridocorneal angle. (**A**) *Angpt4*-expressing cells visualized in cell fate–tracing *Angpt4*^+/Cre^*;Rosa26*^mTmG/+^ mice. *Angpt4*^Cre^-expressing cells are permanently marked with GFP (*green*), while *Angpt4*^Cre^-negative cells express mTomato (*red*). In corneal limbus whole mounts at P10, there are only very few GFP^+^ cells near the SC, while at P13, their number has increased. Areas framed with dashed lines are magnified in the *boxes* below. (**B**) The number of GFP^+^ cells further increases in adult corneal limbus area. SC is stained with CD31 (*white*). Mo, mouse age in months. (**C**) In sectioned tissue, GFP^+^ cells can be more precisely localized into the TM (*arrows*) and into the corneal stroma and sclera (*arrowheads*). *Open arrowheads* point to the GFP^+^ retinal (R) astrocytes. Area framed with *dashed lines* is magnified in the *box* on the *left*. *Arrows* in the *insert* point to projections of which cells of the narrow JCT region just beneath the SC make toward the SC endothelium (*sparse dashed lines*). CB, ciliary body; LVP, limbal vascular plexus. (**D**) *Angpt4* expression visualized in the *Angpt4*^LacZ^ reporter mice with X-gal staining (*blue precipitate, arrowheads*) at P19. (**E**–**H**) qPCR analysis of *Tie2* (**E**), *Angpt1* (**F**), *Angpt2* (**G**), and *Angpt4* (**H**) mRNA expression in the anterior eye (cornea and limbus) of different-aged WT mice normalized to 1-month-old mice. *n* = 4 mice/age group. *P < 0.05, **P < 0.01, ***P < 0.001 in one-way ANOVA followed by Tukey post hoc test.

To characterize the potential role of Angpt4 in the AH drainage and SC, we first investigated its expression in the iridocorneal angle. By crossing the genetic cell fate mapping mouse line *Rosa26*^mTmG^ with *Angpt4*^Cre^ mice,^23^^,^^38^ we were able to visualize the cells that express or have expressed Cre recombinase under the *Angpt4* promoter by Cre-mediated conversion of continuous tdTomato expression to membrane-targeted green fluorescence protein (GFP) expression. We observed that the *Angpt4*-driven GFP signal started postnatally: at P5 (postnatal day 5) and P10, flat-mounted corneal limbus samples had only very few, faint GFP^+^ cells, while at P13, their number and signal intensity were beginning to multiply (Fig. 1A, Supplementary Fig. S1A). GFP^+^ cell number further increased in adult corneolimbal areas (Fig. 1B, Supplementary Figs. S1B–D). Animation of consecutive optical sections acquired by confocal microscopy taken across one such corneal limbus whole mount demonstrated that GFP^+^ cells did not overlap with the SC endothelium, as judged by the lack of merging immunofluorescence signal, but the cells located intimately adjacent to SC outer and inner walls and on the scleral stroma (Supplementary Video S1). Based on tissue cross sections, GFP^+^ cells were more specifically localized into the TM, both into the juxtacanalicular tissue (JCT) directly adjacent to the SC inner wall, as well as into the uveal and corneoscleral meshworks next to the anterior chamber (Fig. 1C, Supplementary Fig. S1E). Cells of the JCT can make bridging contacts with processes extending to the SC endothelium,^12^ and some of the GFP^+^ cells appeared to make such connections (Fig. 1C). Occasional GFP^+^ cells were also seen residing near the SC outer wall endothelium and, more distantly to the SC, embedded into the corneal stroma and the sclera (Fig. 1C). Additionally, arterial smooth muscle cells (Supplementary Fig. S1C) and retinal astrocytes (Fig. 1C) were GFP^+^, as we have reported earlier.^23^^,^^38^

As the cells remain permanently labeled with GFP even after discontinued *Angpt4*^Cre^ expression in the *Rosa26*^mTmG^ model, we characterized expression of *Angpt4* in selected time points by using *Angpt4*^LacZ^ mice, which express the *LacZ* gene encoding β-galactosidase enzyme under the *Angpt4* promoter.^23^ X-gal–stained *Angpt4*^LacZ/LacZ^ eyes had a few cells around the SC with very subtle blue staining at P10 and more intense blue staining in individual cells in the TM at P13, P19, 2 months, and 9 months of age (Fig. 1D, Supplementary Fig. S2). This confirmed the expression pattern of Angpt4 adjacent to the SC, as observed in the *Angpt4*^Cre^*;Rosa26*^mTmG^ model, and suggested that Angpt4 is constantly produced at moderate levels in the adult TM.

To further examine the expression of Angpts, Tie receptors, and VE-PTP in the mouse iridocorneal angle cells, we utilized the previously published, openly available scRNAseq data set that has been collected from 12-week-old CD1 mice.^4^ scRNAseq data essentially confirmed our findings in the reporter mouse lines by showing *Angpt4* expression in the TM (Supplementary Fig. S3). Following the original cell clustering by van Zyl et al.,^4^ beam A and beam Y clusters represent the filtering TM cells from the uveal and corneoscleral meshworks, and the JCT forms its own cluster. *Angpt4* expression was found in the JCT and beam A cells as well as in the corneal and uveal clusters, which likely represent the GFP^+^ cells seen in the corneal/scleral stroma (Supplementary Fig. S3). In line with previous reports,^16^^,^^17^^,^^26^ *Angpt1* was expressed in all TM clusters (Supplementary Fig. S3), including *Angpt4*-negative beam Y cells. In contrast to *Angpt1* and *Angpt4*, *Angpt2* was robustly expressed in the corneal endothelium and, to a lesser extent, also in the SC endothelium and vascular endothelium (Supplementary Fig. S3).^16^^,^^26^ *Angpt2* expression was also found in the JCT but notably not in any of the beam cell types of the TM that expressed *Angpt1* and *Angpt4* (Supplementary Fig. S3). *Tie1*, *Tie2* (*Tek*), and *Ptprb* (protein tyrosine phosphatase receptor type B encoding VE-PTP) were all expressed in the *Angpt4*-negative vascular and SC endothelial cells (Supplementary Fig. S3), as has been reported.^17^^,^^26^^,^^30^^,^^31^ In addition, some of the *Tie2*^+^ cells mapped to the beam Y cluster (Supplementary Fig. S3). As *Angpt1* and *Angpt4* were partly expressed in the same cell types, the prevalence of their co-occurrence was further investigated. As indicated in Supplementary Table S2, among the *Angpt4*^+^ cells, *Angpt1* was most frequently simultaneously expressed in the corneal epithelial and uveal cells, whereas, for example, *Angpt4*^+^ beam A cells were distinct from the *Angpt1*^+^ beam A cells.

Finally, qPCR analysis of anterior eye tissues from WT mice (including both the cornea and the limbus area) was used to determine an individual expression profile over time for the *Angpts* and *Tie2* (Figs. 1E–H). *Tie2*, *Angpt1*, and *Angpt2* mRNA levels were the highest in the early postnatal days (P10) and were thereafter downregulated, in line with previously reported results.^14^^,^^26^ *Angpt2* expression was downregulated more gradually than *Angpt1* (Figs. 1F–G) while *Angpt4* expression was overall rather steady, with a slight peak at 1 month of age (Fig. 1H). This was in line with the results obtained from the *Angpt4*^Cre^*;Rosa26*^mTmG^ and *Angpt4*^LacZ^ mice and suggested that *Angpt4* mRNA is already expressed at P10, a few days prior to significant reporter protein detection in the microscopic analyses.

Collectively, spatiotemporal gene expression data from the cell fate tracing and reporter mice, scRNAseq, and qPCR analyses indicated that Angpt4 is expressed at relatively low levels in the TM cells close to the SC. In our analyses, Angpt4 was not enriched during the postnatal SC development and was partly produced in the same cell types than Angpt1 in adult mice, and the cellular sources of Angpt4 were markedly different from those of Angpt2.

### *Angpt4* Deletion Alone Does Not Affect SC Area or Exacerbate the Effects of *Angpt1* Deletion on SC Maintenance

**Figure 2.**
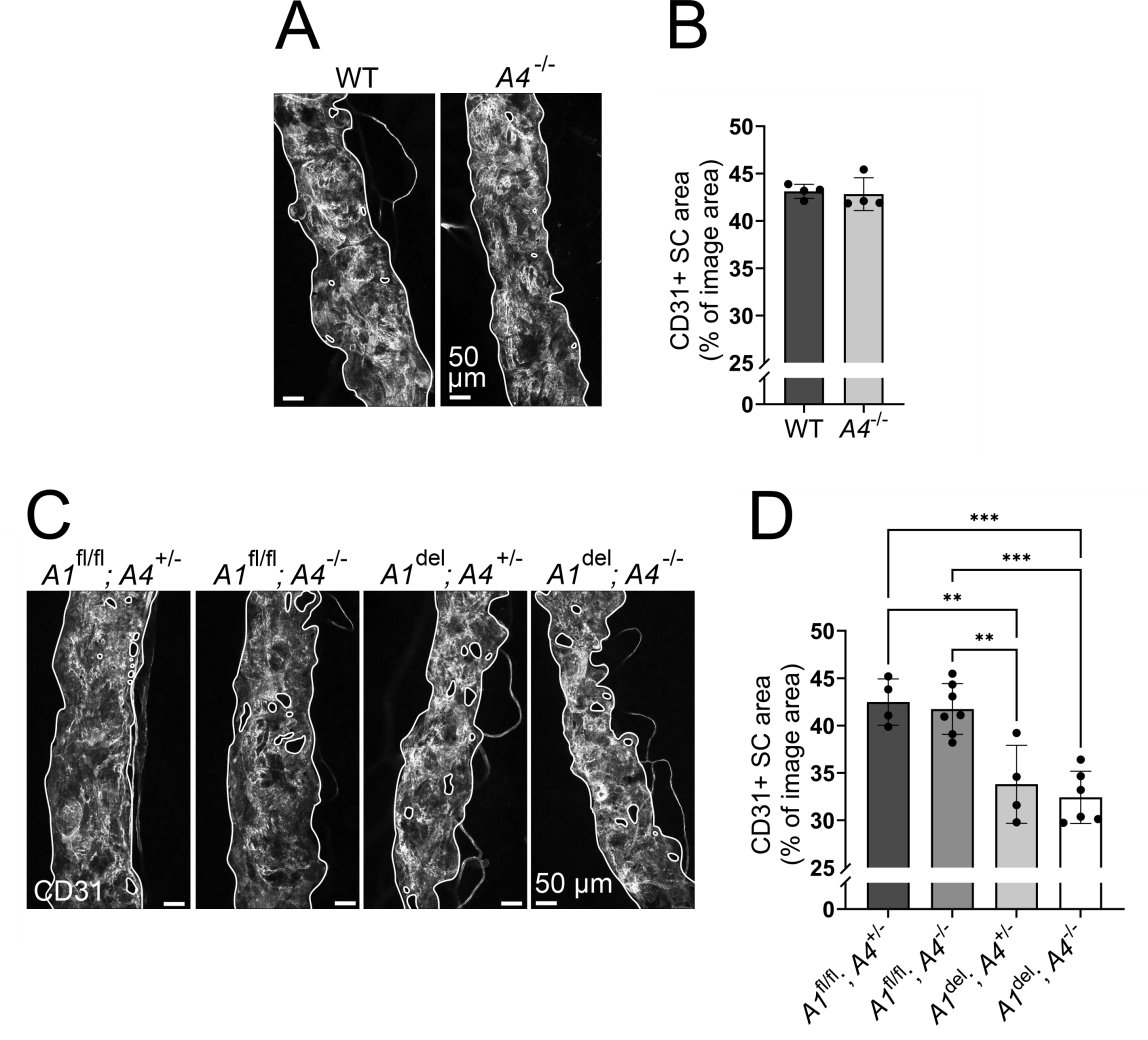
Analyses of the SC area in 1-year-old *Angpt4*-deficient and *Angpt1;Angpt4* double-deficient mice. (**A**) Representative closeup images of CD31 antibody staining (*white*) of whole-mount SC from WT and *Angpt4*^−/−^ (*A4*^−/−^) mice. SC area (highlighted with *solid white lines*) was quantified in (**B**). *n* = 4 mice/genotype. (**C**) Ubiquitous *Angpt1* deletion in *Angpt1*^fl/fl^*;Angpt4*^Cre/Cre^*;Rosa26*^CreERT2^ mice (*A1*^del^; *A4*^−/−^ mice) was induced by weekly oral administration of tamoxifen at 4 to 8 weeks of age. Littermates received the same treatments (*A4*^+/−^ mice are phenotypically normal). Representative closeup images of CD31 antibody staining (*white*) of whole-mount SC are shown. SC area (highlighted with *solid white lines*) was quantified in (**D**). *n* = 4/7/4/6 mice/genotype. **P < 0.01, ***P < 0.001 in one-way ANOVA followed by Tukey post hoc test in **D**.

Motivated by the observed Angpt4 expression adjacent to the SC, we next studied the effect of *Angpt4* deletion on the SC. Since prominent Angpt4 expression appeared postnatally at a time point when the SC is already mostly developed (develops by P9, matures by P20),^13^^–^^15^ we focused on investigating the SC maintenance in adult mice rather than the earlier developmental stages, also keeping in mind possible compensatory/complementary roles of the other Angpts as previously observed in the case of Angpt1 and Angpt2.^16^^,^^24^^,^^26^ SC area analysis from flat-mounted, immunostained corneal limbus samples revealed no changes in the CD31^+^ SC area in 1-year-old *Angpt4*^Cre/Cre^ (hereafter *Angpt4*^−/−^) mice (Figs. 2A, 2B), indicating that Angpt4 is dispensable for SC maintenance. To study the possible combined effects of *Angpt1* and *Angpt4* deletion on SC maintenance, we crossed floxed *Angpt1* mice with *Angpt4*^−/−^ and *Rosa26*^CreERT2^ mice, induced ubiquitous *Angpt1* deletion starting at 4 weeks of age by oral tamoxifen administration, and analyzed the mice at the age of 1 year (Supplementary Fig. S4A). qPCR confirmed proper *Angpt1* deletion efficiency (Supplementary Fig. S4B). As expected, SC area was reduced and SC narrowing point count increased (indicating impaired AH flow) in both *Angpt1*^del^*;Angpt4*^+/−^ and *Angpt1*^del^*;Angpt4*^−/−^ mice when compared to control littermate mice without the *Rosa26*^CreERT2^ allele (Figs. 2C, 2D; Supplementary Figs. S4C, S4D), but no additional severity was detected in the double knockout mice compared to single *Angpt1* knockout mice. Neither collecting vein branch number count, corneal arcade count (loops of the limbal capillary plexus toward the corneal side), existence of circulating limbal arteries and veins used as a measure of complexity of the limbal vasculature,^40^ nor Lyve1^+^ corneolimbal LV area were changed in any of the genotypes (Supplementary Figs. S4C, 4E–G). In line with the SC area defects, IOP was elevated in the *Angpt1*^del^ mice regardless of *Angpt4* deletion (Supplementary Fig. S4H). Hence, our results indicated that additional *Angpt4* deletion does not aggravate the phenotype seen in the *Angpt1* knockout mice.

### Combined Deletion of *Angpt2* and *Angpt4* Causes Severe Defects in SC Morphology

In recent GWAS analyses, *ANGPT2* has been identified as a risk locus associated with glaucoma,^5^^–^^8^ but the importance of Angpt2 for the SC has not been unanimously shown, and no congenital *Angpt2* deletion model has been used in any of the previous mouse studies.^16^^,^^26^^,^^32^ Therefore, to generate a germline loss-of-function *Angpt2* allele (*Angpt2*^−/−^ mice), we utilized the CRISPR/Cas9 methodology to induce a 35-bp deletion in the *Angpt2* exon 1. This resulted in a codon frameshift and decreased *Angpt2* mRNA expression, and Angpt2 antibody staining confirmed the absence of Angpt2 protein (Supplementary Fig. S5). To assess the possible additive effects of Angpt2 and Angpt4, *Angpt2*^−/−^ mice were crossed with *Angpt4*^−/−^ mice to generate double knockout mice, and SC morphology was investigated in detail in WT, *Angpt2*^−/−^, *Angpt4*^−/−^, and *Angpt2*^−/−^*;Angpt4*^−/−^ mice.

**Figure 3.**
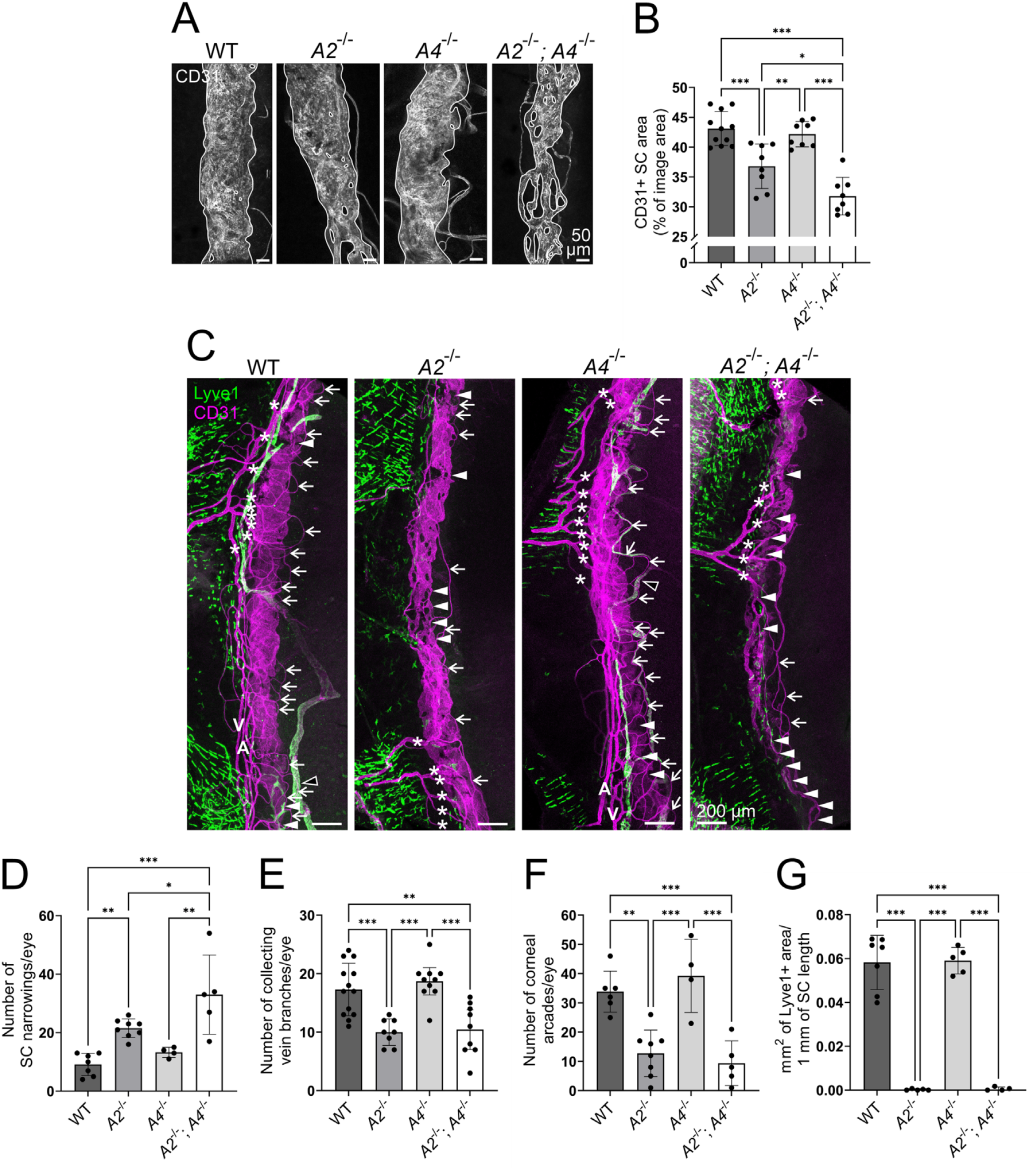
Analyses of SC morphology in 1-year-old *Angpt2*^−/−^ and *Angpt2*^−/−^*;Angpt4*^−/−^ mice. (**A**) Representative closeup images of CD31 antibody staining (*white*) of whole-mount SC. SC area (highlighted with *solid white lines*) was quantified in (**B**). *n* = 11/8/8/8 mice/genotype. (**C**) Representative images of whole-mount corneal limbus area. CD31 (*magenta*) strongly labels the SC and limbal blood vasculature and more faintly the LVs, and Lyve1 (*green*) distinctly marks LVs (*open arrowheads*) and also limbal macrophages. A, circular limbal artery; V, perilimbal vein. *Arrowheads* mark SC narrowing points quantified in (**D**), *asterisks* mark collecting vein branches quantified in (**E**), *arrows* mark corneal arcades of the limbal capillary plexus quantified in (**F**), and Lyve1^+^ corneolimbal LVs were quantified in (**G**). *n* = 7/8/4/5 mice/genotype in **D**, *n* = 13/8/10/9 mice/genotype in **E**, *n* = 6/8/4/5 mice/genotype in **F**, and *n* = 7/5/5/4 mice/genotype in **G**. *P < 0.05, **P < 0.01, ***P < 0.001 in one-way ANOVA followed by Tukey post hoc test in **B** and **D**–**F** or in Welch's ANOVA followed by Dunnett's T3 post hoc test in **G**.

At 1 year of age, *Angpt2*^−/−^ mice had a decreased SC area and increased SC narrowing count when compared to WT and *Angpt4*^−/−^ mice (Figs. 3A–D). Notably, *Angpt2*^−/−^*;Angpt4*^−/−^ mice had a smaller SC area and more SC narrowings than any of the other genotypes (Figs. 3A–D). The limbal vascular plexus in *Angpt2*^−/−^ and *Angpt2*^−/−^*;Angpt4*^−/−^ mice was also less complex with loss of typical circular limbal arteries and perilimbal veins^40^ (Fig. 3C). Clearly distinguishable patterns of circulating parallel limbal arteries and veins described in mice with partial C57BL/6 background^40^ were detected in 7/7 WT and 4/4 *Angpt4*^−/−^ samples, but such arteries were very rarely seen when *Angpt2* was deleted (in 2/8 *Angpt2*^−/−^ and 0/5 *Angpt2*^−/−^*;Angpt4*^−/−^ samples), and no circulating veins were found when 8 *Angpt2*^−/−^ and 5 *Angpt2*^−/−^*;Angpt4*^−/−^ samples were analyzed (Fig. 3C). Additionally, the collecting veins (determined as the branching venous plexus and collector channels enriched at the sites of episcleral and aqueous veins in comparison to the above-discussed perilimbal veins that circulate alongside the SC) and the corneal arcades of the capillary plexus appeared to be dependent on Angpt2 as both *Angpt2*^−/−^ and *Angpt2*^−/−^*;Angpt4*^−/−^ mice displayed similar reduction in collecting vein branch and corneal arcade number (Figs. 3E, 3F). Both *Angpt2*^−/−^ and *Angpt2*^−/−^*;Angpt4*^−/−^ mice also completely lacked corneolimbal LVs while WT and *Angpt4*^−/−^ mice had no changes (Fig. 3G), along with a previous study reporting reduced LV area in a postnatally induced *Angpt2* deletion model^26^ and implying that Angpt2 is indispensable for the development of both limbal blood and lymphatic vasculature. The observed vascular phenotypes in the *Angpt2*-deficient mice were consistent with the scRNAseq data, and additionally, immunostaining localized Angpt2 expression to the LVs (Supplementary Fig. S6). Essentially similar, albeit slightly less pronounced, SC and corneolimbal vasculature phenotypes were also seen in 12-week-old *Angpt2*^−/−^ and *Angpt2*^−/−^*;Angpt4*^−/−^ mice (Supplementary Fig. S7). All in all, our data imply that *Angpt2* is required for the proper SC and corneolimbal blood and lymphatic vasculature development, and additional *Angpt4* deletion aggravates the negative impact of *Angpt2* deletion on the SC phenotype.

### Aged *Angpt2*^−/−^*;Angpt4*^−/−^ Mice Have Glaucoma-Associated Pathologic Changes

**Figure 4.**
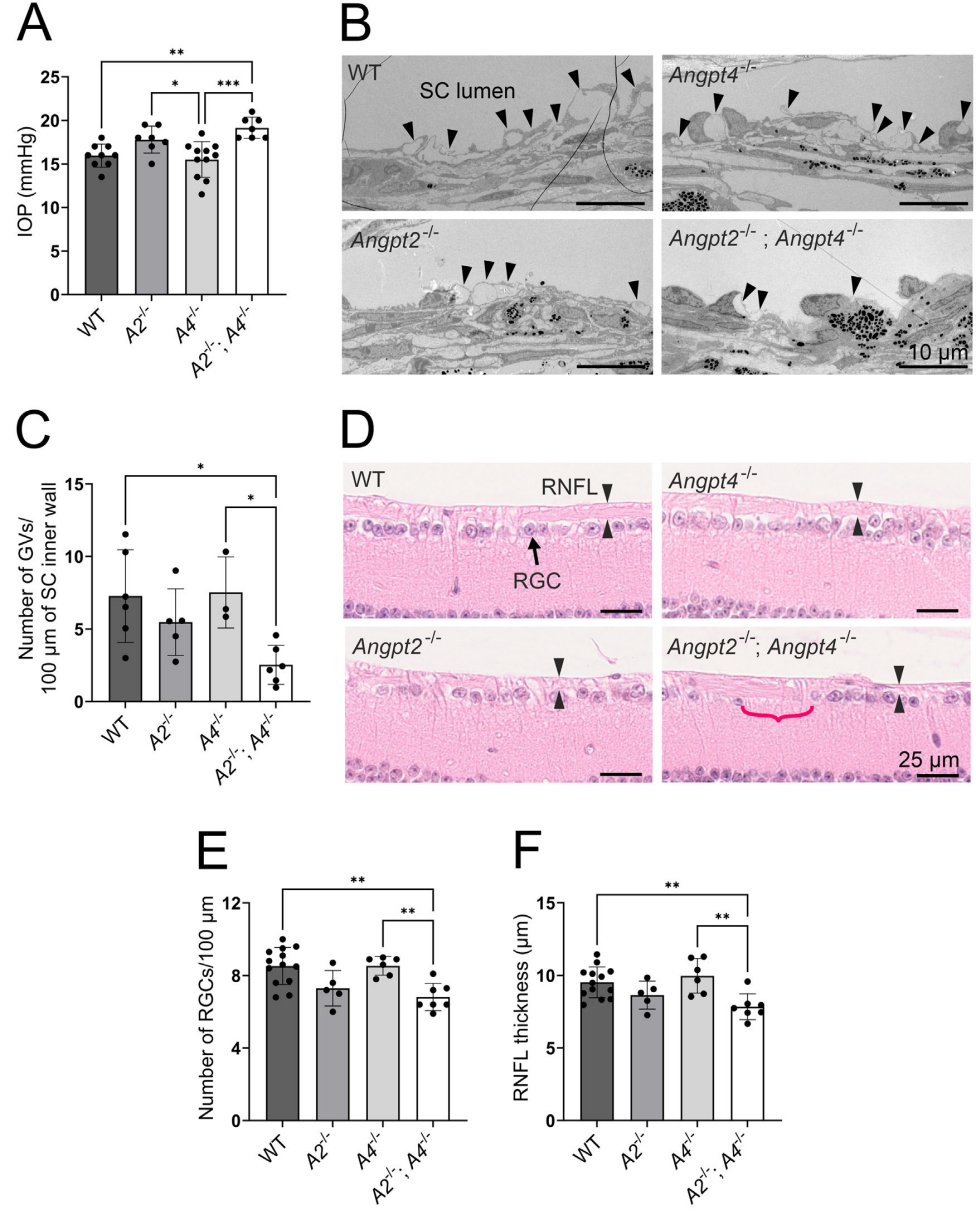
Analysis of glaucoma-like changes in 1-year-old *Angpt2*^−/−^ and *Angpt2*^−/−^*;Angpt4*^−/−^ mice. (**A**) IOP measurements. *n* = 9/7/11/7 mice/genotype. (**B**, **C**) Transmission electron microscopy images (**B**) were used to analyze GV number (*arrowheads*) in the SC inner wall endothelium in (**C**). *n* = 6/5/3/6 mice/genotype. (**D**) Hematoxylin and eosin–stained sections were used to analyze RGC (*arrow*) number (**E**) and RNFL (*opposite arrowheads*) thickness (**F**). *Red bracket* highlights RGC-deprived area in the *Angpt2*^−/−^*;Angpt4*^−/−^ mice. *n* = 13/5/6/7 mice/genotype. *P < 0.05, **P < 0.01, ***P < 0.001 in one-way ANOVA followed by Tukey post hoc test.

Based on the defects in the SC morphology, we next investigated whether *Angpt2*^−/−^ and *Angpt2*^−/−^*;Angpt4*^−/−^ mice have glaucoma-associated functional defects as well. IOP was significantly elevated in the aged *Angpt2*^−/−^*;Angpt4*^−/−^ mice (Fig. 4A). Expression of lymphatic transcription factor Prox1, which is essential for SC integrity and a functional marker of proper AH outflow,^14^ was slightly decreased in the *Angpt2*^−/−^*;Angpt4*^−/−^ mice (Supplementary Fig. S8). We also further examined the ultrastructure of SC endothelium for signs of weakened flow. Giant vacuoles (GVs) are outpouchings of the SC inner wall endothelial cells involved in AH outflow from the JCT to the SC, and their amount is decreased in response to poor AH flow.^26^^,^^41^^,^^42^ We found reduced number of GVs only in aged *Angpt2*^−/−^*;Angpt4*^−/−^ mice when compared to WT and *Angpt4*^−/−^ mice (Figs. 4B, 4C). As an implication of neuronal damage, we studied the retinal histology of the aged mice. The number of retinal ganglion cells (RGCs) and thickness of the retinal nerve fiber layer (RNFL) were significantly reduced only in *Angpt2*^−/−^*;Angpt4*^−/−^ mice (Figs. 4D–F), although *Angpt2*^−/−^ mice displayed a similar trend as well. Collectively, these data showed that *Angpt2*^−/−^*;Angpt4*^−/−^ mice exhibit a glaucoma-like phenotype.

### Angpt2 and Angpt4 Cooperate as Tie2 Agonists in SC Maintenance

**Figure 5.**
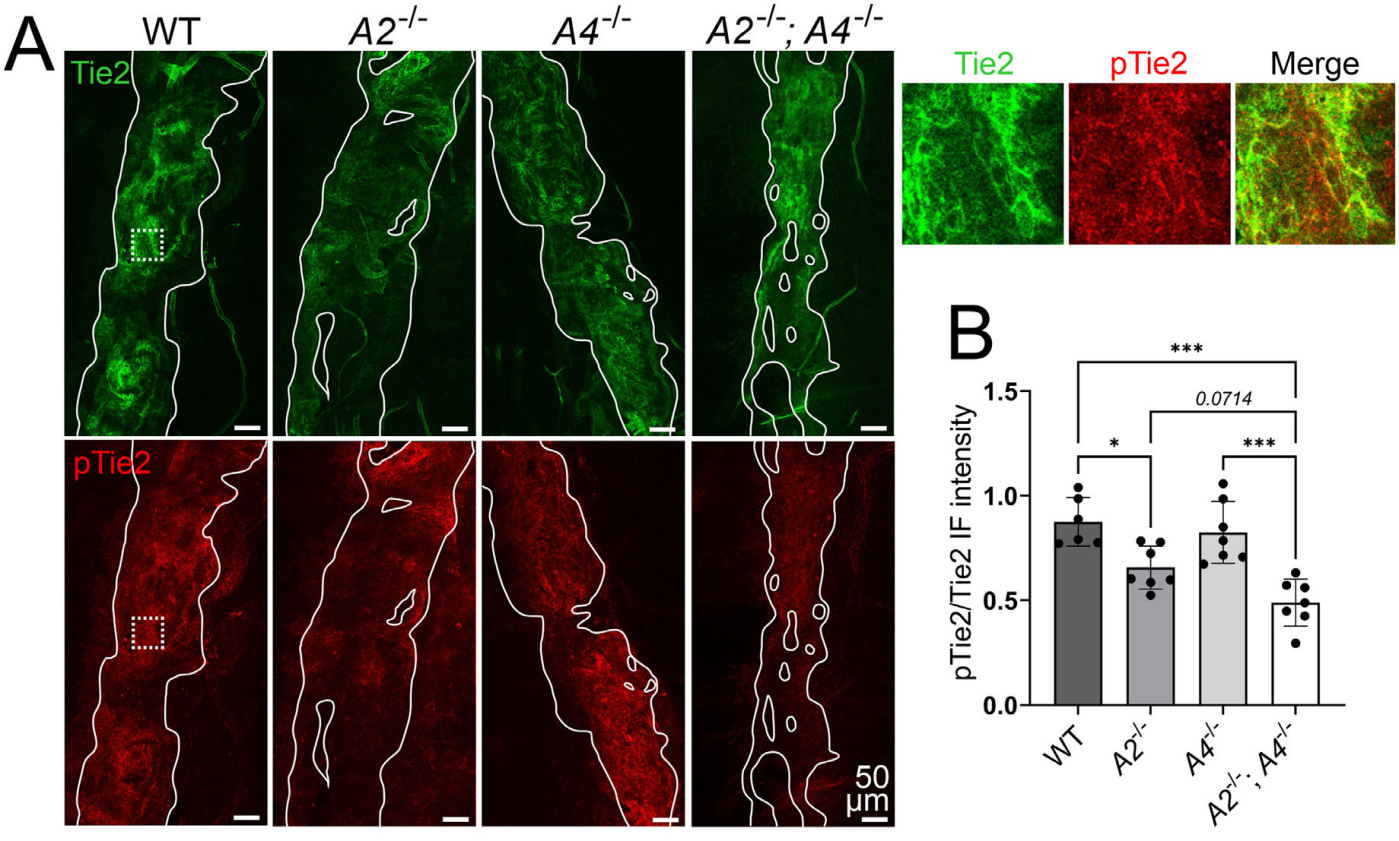
Analysis of Tie2 activation in the SC endothelium of *Angpt2*^−/−^ and *Angpt2*^−/−^;*Angpt4*^−/−^ mice. (**A**) Representative images of SC (highlighted with *solid white lines*) from 12-week-old mice stained with total Tie2 (*green*) and phosphorylated Tie2 (pTie2; *red*). Areas separated with *dashed lines* are magnified in the small boxes. (**B**) Quantification of pTie2/Tie2 immunofluorescence intensity ratio in the SC. *n* = 6–7 mice/genotype. *P < 0.05, ***P < 0.001 in one-way ANOVA followed by Tukey post hoc test.

To investigate the possible Tie2-dependent mechanism of cooperative action of Angpt2 and Angpt4 on the SC, we performed antibody staining using phospho-Tie2 (pTie2) antibody that has previously been used to indicate Tie2 activation state in the SC.^26^^,^^30^ pTie2 was studied from younger mice (12 weeks of age) as total Tie2 and pTie2 levels have been shown to markedly decrease during aging^26^ and the 12-week-old mice already showed a similar SC phenotype than the older mice (Supplementary Fig. S7). In the *Angpt2*^−/−^ mice, pTie2/Tie2 ratio decreased, indicating that Angpt2 functions as a Tie2 agonist in the SC (Figs. 5A, 5B). Single *Angpt4* deletion did not significantly affect Tie2 phosphorylation (Figs. 5A, 5B), but intriguingly, deletion of both *Angpt2* and *Angpt4* appeared to reduce the pTie2/Tie2 ratio more than single *Angpt2* deletion (Figs. 5A, 5B). When comparing all four groups in one-way ANOVA, the *P*-value of 0.07 was just above the 0.05 threshold; however, when comparing *Angpt2*^−/−^ mice to *Angpt2*^−/−^*;Angpt4*^−/−^ mice with an unpaired Student's two-tailed *t*-test, *Angpt2*^−/−^*;Angpt4*^−/−^ mice showed a statistically significantly reduced pTie2/Tie2 ratio (*P* = 0.022).

**Figure 6.**
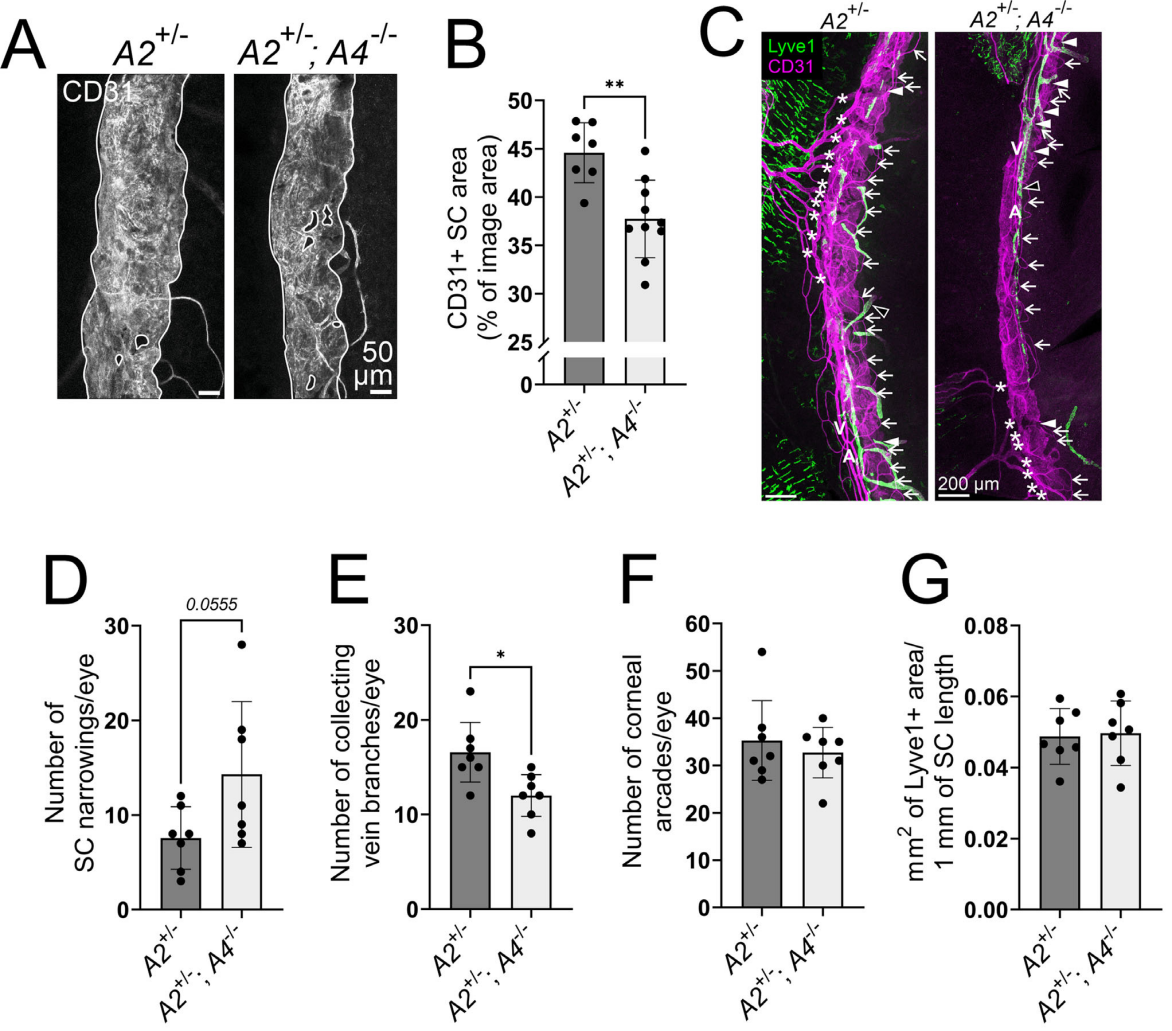
Analyses of SC morphology in 1-year-old *Angpt2*^+/−^ and *Angpt2*^+/−^*;Angpt4*^−/−^ mice. (**A**) Representative closeup images of CD31 antibody staining (*white*) of whole-mount SC. SC area (highlighted with *solid white lines*) was quantified in (**B**). *n* = 7/10 mice/genotype. (**C**) Representative images of whole-mount corneal limbus area. CD31 (*magenta*) strongly labels the SC and limbal blood vasculature and, more faintly, the LVs, and Lyve1 (*green*) distinctly marks the LVs (*open arrowheads*) and also limbal macrophages. A, circular limbal artery; V, perilimbal vein. *Arrowheads* mark SC narrowing points quantified in (**D**), *asterisks* mark collecting vein branches quantified in (**E**), *arrows* mark corneal arcades of the limbal capillary plexus quantified in (**F**), and Lyve1^+^ corneolimbal LVs were quantified in (**G**). *n* = 7 mice/genotype in **D**–**G**. *P < 0.05, **P < 0.01 in unpaired Student's two-tailed *t*-test.

**Figure 7.**
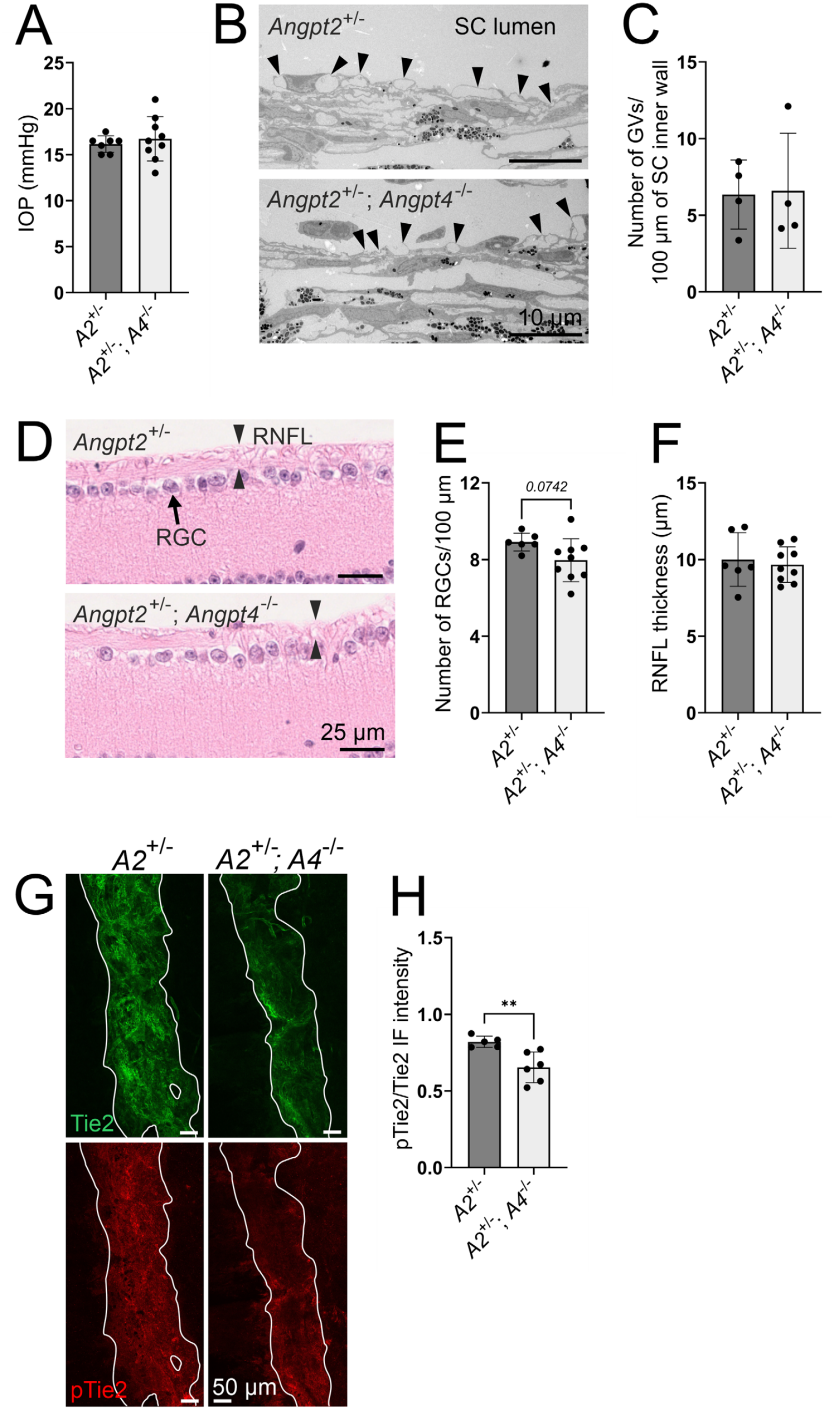
Analyses of glaucoma-like changes and Tie2 activation in 1-year-old *Angpt2*^+/−^ and *Angpt2*^+/−^*;Angpt4*^−/−^ mice. (**A**) IOP measurements. *n* = 7/9 mice/genotype. (**B**, **C**) GV number (*arrowheads*) in the SC inner wall endothelium was analyzed from transmission electron microscopy images (**B**) and quantified in (**C**). *n* = 4 mice/genotype. (**D**–**F**) RGC (*arrow*) number and RNFL (*opposite arrowheads*) thickness was quantified from hematoxylin and eosin–stained sections. *n* = 6/9 mice/genotype. (**G**) Representative images of SC (highlighted with *solid white lines*) stained with total Tie2 (*green*) and phosphorylated Tie2 (pTie2, *red*). (**H**) Quantification of pTie2/Tie2 immunofluorescence intensity ratio in the SC. *n* = 5/6 mice/genotype. **P < 0.01 in unpaired Student's two-tailed *t*-test.

Our results suggested that Angpt4 contributes to the dose-dependent Tie2 activation in the SC. To confirm this, we investigated Angpt4 deficiency in a potentially more sensitive genetic combination without the strong overall effects of total *Angpt2* deletion by comparing *Angpt2*^+/−^ and *Angpt2*^+/−^*;Angpt4*^−/−^ mice. While at 12 weeks of age, these mice did not have major differences in the SC morphology (Supplementary Fig. S9), 1-year-old *Angpt2*^+/−^*;Angpt4*^−/−^ mice showed significantly reduced SC area (Figs. 6A, 6B), further indicating a role for Angpt4 in the maintenance of the SC. *Angpt2*^+/−^*;Angpt4*^−/−^ mice had only a trend toward an increase in SC narrowing points (Figs. 6C, 6D), but rather surprisingly, collector vein branching was decreased in *Angpt2*^+/−^*;Angpt4*^−/−^ mice (Fig. 6E). However, no differences in the corneal arcades, circulating limbal arteries and veins (7/7 *Angpt2*^+/−^ and 7/7 *Angpt2*^+/−^*;Angpt4*^−/−^ samples had both), or corneolimbal LVs were seen (Figs. 6C, 6F–G), and *Angpt2*^+/−^ mice had limbal LV area comparable to WT mice (*P* = 0.11 in unpaired two-tailed Student's *t*-test). Despite the reduction in SC area, aged *Angpt2*^+/−^*;Angpt4*^−/−^ mice did not display major signs of glaucoma (Figs. 7A–F), possibly owing to their less convoluted and thus less obstructive SC morphology when compared to full *Angpt2* deletion mice. Nonetheless, the pTie2/Tie2 ratio was reduced in *Angpt2*^+/−^*;Angpt4*^−/−^ SC endothelium when compared to *Angpt2*^+/−^ mice (Figs. 7G–H), indicating that also Angpt4 is a Tie2 agonist in the SC and supports optimal SC structure and drainage function.

## Discussion

The requirement of receptor tyrosine kinase Tie2 activation for SC morphogenesis and maintenance of fluid homeostasis in the anterior eye has been well established.^16^^,^^17^^,^^24^^–^^30^ However, the contributions of all three Tie2 ligands are not fully elucidated: the importance of Angpt4 has not been previously investigated, and the exact role of Angpt2 has remained controversial. For the first time, our data reveal the spatiotemporal expression pattern of Angpt4 in the close vicinity of the SC, as well as the physiologic importance of Angpt4 and its cooperation with Angpt2 in maintaining AH drainage. Our results also support the necessity of Angpt2 for the formation of functionally competent SC and corneolimbal vasculature in mice.

An outstanding question regarding the Angpt/Tie2-dependent development and maintenance of the SC is the evident presence of three Angpt ligands to activate the Tie2 receptor in the same vascular structure. According to our data presented in this study and the results of others, Angpt1 is highly expressed at the time of SC development and at lower levels later in life,^17^^,^^26^ suggesting an important role in initial SC formation. This is in line with severe SC defects in mice with induced deletion of *Angpt1* at E16.5 (50–70% decrease in SC area)^16^^,^^24^ or at P1–P5 (50% decrease in SC area)^26^ and with the lack of such a phenotype when *Angpt1* is deleted at 8 weeks of age.^26^ In our experimental setting (to investigate Angpt1 interplay with postnatally expressed Angpt4), *Angpt1* deletion was induced at 4 weeks of age. This was sufficient to cause a hypomorphic SC and an increase in IOP, but the defects were not as severe as in the abovementioned earlier time-point deletions by others (25% decrease in SC area in our model). Altogether, these studies indicate that SC maintenance is less dependent on Angpt1 and that other Tie2 ligands may be more important for that. In this regard, it was interesting to find that both Angpt1 (our data and previously reported)^16^^,^^17^^,^^26^ and Angpt4 (our data) are expressed in the same tissue compartment, the filtrating TM, which may serve as a reservoir of Angpt1 and Angpt4 to activate Tie2 at the SC endothelium.^17^ This type of paracrine signaling is common in the Angpt/Tie2 pathway and occurs, for example, between Purkinje, endothelial, and neural cells.^43^ scRNAseq data from adult mice revealed that some TM cells had overlapping expression of both Angpt1 and Angpt4, whereas most TM cells expressed only either one (e.g., among the Angpt1^+^ JCT cells, 10.5% simultaneously expressed Angpt4, being 50% of Angpt4^+^ JCT cells). The lack of any SC defect in the *Angpt1*^fl/fl^*;Angpt4*^Cre/Cre^ mice, in which *Angpt4* promoter–driven Cre expression (starting at P10) would prevent Angpt1 expression from the floxed allele if occurring in the same cells, also supports this observation.

In contrast to Angpt1 and Angpt4, Angpt2 was in our studies rather steadily present throughout the lifetime in almost all iridocorneal angle structures, but Angpt2 expression was detected only in the JCT but not in the other TM compartments. We showed that Angpt2 is expressed and essential in the limbal blood, lymphatic, and SC endothelium, suggesting that Angpt2 may also activate Tie2 in an autocrine manner, thereby mechanistically differing from the other Angpt ligands in the iridocorneal angle vascular structures. The notion that *Angpt4* deletion exacerbated the phenotype of *Angpt2* deletion but not *Angpt1* deletion might be explained by the result that Angpt2 and Angpt4 are produced in different cellular compartments, making their combination deletion more harmful and difficult to compensate by the remaining ligand Angpt1. In addition, as Angpt1 is the high-affinity and strongly Tie2-phosphorylating agonistic ligand, combination deletion with Angpt4, which is a weaker ligand^23^ and not as strongly expressed in the iridocorneal angle, may not be potent enough to cause substantial additional effect.

So far, reports of the effect of *Angpt2* deficiency on AH drainage have been conflicting, either showing no effect at all^16^^,^^32^ or resulting in reduced SC area^26^ and aggravated AH pathologies when combined with *Angpt1* deficiency.^16^^,^^24^^,^^26^ Our study clearly indicates that germline *Angpt2* deletion alone is sufficient to cause defects in the SC in mice, an observation that goes hand in hand with GWAS reports associating *ANGPT2* genetic variants with patients who have increased IOP and glaucoma.^5^^–^^8^ Previous negative findings have been done in inducible gene deletion models or after injecting Angpt2 blocking antibodies that both may result in incomplete Angpt2 inhibition. In addition, variations in the deletion and analysis time points, model species, and genetic mouse backgrounds may explain some of the observed differences. *Angpt2*^−/−^ mice are also known to have abnormal retinal vasculature with sprouting defects and persistent hyaloid vessels,^35^^,^^44^^,^^45^ and *Angpt4*^−/−^ mice have impaired retinal vein development, leading to inner nuclear layer swelling.^23^ The influence of the defective retinal vasculature on RGC count/RNFL thickness in these mice cannot be fully excluded, but insufficient AH outflow was functionally evident as an increase in IOP and also more indirectly suggested by the reduction in Prox1 expression and GV number in the *Angpt2*^−/−^*;Angpt4*^−/−^ mice.

Our data intriguingly showed that certain SC phenotypes were more severe in the double-deficient *Angpt2*^−/−^*;Angpt4*^−/−^ mice than in the *Angpt2*^−/−^ mice, correlating with the slightly diminished Tie2 phosphorylation state in the SC endothelium. Strikingly, also *Angpt2*^+/−^*;Angpt4*^−/−^ mice, unlike *Angpt2*^+/−^ or *Angpt4*^−/−^ mice, had a decreased SC area and Tie2 phosphorylation state, providing further evidence of Angpt4 as a regulator of the SC. The herein proposed model of Angpt2 and Angpt4 acting as Tie2 agonists in the SC endothelium correlates with the established role of Angpt2 as a Tie2 agonist in the lymphatic endothelium^21^ and the reported modest and somewhat patchy VE-PTP expression in the mouse SC, especially at the inner wall.^17^^,^^30^ Along the SC, *Angpt2* deletion dramatically affected certain areas while other portions were unaffected, suggesting that local changes in the availability of the relatively weaker Tie2 ligands Angpt2 and Angpt4 could also fine-tune Tie2 activation state, providing homeostatic support for the SC after its developmental phase mainly mediated by the strongest Tie2 ligand Angpt1. Considering previous publications^16^^,^^24^^,^^26^ demonstrating the additive effect of Angpt1 and Angpt2 on SC development and our findings in *Angpt2*^−/−^*;Angpt4*^−/−^ mice, it can be concluded that Tie2-dependent AH homeostasis is dependent on a sufficiently high amount of Angpt ligands.

Intriguingly, our data also revealed qualitative differences between the genotypes investigated. In *Angpt2*^+/−^*;Angpt4*^−/−^ mice, the SC was continuously slightly narrower than in WT, *Angpt2*^+/−^, or *Angpt4*^−/−^ mice, but it did not have as much such extremely narrow convolutions as the SC in *Angpt2*^−/−^ and *Angpt2*^−/−^*;Angpt4*^−/−^ mice had, suggesting that Angpt2 is essential for adequate SC morphogenesis, but also Angpt4 contributes to the maintenance of optimal width of the mature canal. Despite the reduction in total SC area and pTie2/Tie2 ratio comparable to *Angpt2*^−/−^ mice, *Angpt2*^+/−^*;Angpt4*^−/−^ mice did not have elevated IOP or significant RGC damage. Likely, the numerous SC narrowing points and convolutions seen in *Angpt2*^−/−^ and *Angpt2*^−/−^*;Angpt4*^−/−^ mice are obstructive and inhibit the proper flow of AH in the SC more than a steadily narrow but otherwise normal SC does.

As another novel finding, we showed that Angpt2 is required for the formation of proper corneolimbal blood vasculature (collector veins, circulating arteries/veins, and corneal arcades). Interestingly, defects in the limbal vasculature have also been described in *Tie1* and *Tie2* and double *Angpt1;Angpt2* deletion mice^24^^,^^26^^,^^31^ and in mice lacking Svep1, an extracellular matrix protein linked to the Angpt2/Tie pathway as a modulating factor.^17^^,^^28^^,^^46^ In our study, *Angpt1*^del^ mice did not show any apparent defects in the limbal vasculature maintenance, and limbal BV defects have not been reported in the early *Angpt1* deletion models by others, implying that specifically, Angpt2/Tie/Svep1 interplay would be needed in the corneolimbal vasculature. We also found that *Angpt2*^−/−^ mice completely lacked corneolimbal lymphatics, in line with published data on early postnatal deletion of *Angpt2* or *Tie2* resulting in significant but not complete absence of corneolimbal LVs,^26^ suggesting that Angpt2 is required for corneolimbal LV development very early on. Double *Angpt1;Angpt2* deletion mice have also been reported to lack corneolimbal LVs,^24^ but *Angpt1* deletion alone has only minor^26^ or no effect (Thomson et al.^24^ and our data), indicating that Angpt2 is the major Tie2 ligand in the corneolimbal LVs. Currently, however, the significance of the corneolimbal LVs for maintaining AH outflow remains debated.^47^^–^^49^ In our study, the absence (in *Angpt2*^−/−^*;Angpt4*^−/−^) or presence (*Angpt1*^del^) of both corneolimbal BVs and LVs had no obvious additional effects on IOP in models with comparable reduction in SC area.

In conclusion, this is the first study to indicate a role for Angpt4 in the SC maintenance. In addition, we provide evidence of Angpt2 as an essential SC and corneolimbal vasculature regulator. All three angiopoietins, Angpt1, Angpt2, and Angpt4, seem to be derived from varying cellular sources in the iridocorneal angle. Previous studies comparing the primary structures of human and mouse Angpt1, Angpt2, and Angpt4 have revealed that Angpt4 is the least conserved ligand among the angiopoietins.^50^ Furthermore, biochemical characteristics of Angpt1, Angpt2, and Angpt4 are not identical, and they have ligand-specific effects in vitro.^23^ The presence of three different Angpt ligands may be necessary for fine-tuning the SC morphology and functionality: treatment with a designed recombinant protein combining the beneficial characteristics of Angpt ligands or with an Angpt peptide cocktail might produce an effect best resembling the physiologic function of Angpts and result in an efficient therapeutic outcome. All in all, future investigations should exploit the full Angpt/Tie2 pathway when further characterizing SC development and maintenance, mapping risk alleles in patients, and developing new therapeutics against glaucoma. The experimental evidence provided here also further rationalizes the testing of Tie2 activation agents, such as Angpt2-clustering and Tie2-activating antibody ABTAA,^26^ VE-PTP inhibitor AKB-9778,^30^^,^^51^ and hepta-ANGPT1^17^ as possible therapeutic approaches to restore AH drainage function.

## Supplementary Material

Supplement 1

Supplement 2
