# Supplementary material for: Cooperation of Angiopoietin-2 and Angiopoietin-4 in Schlemm's Canal Maintenance
Source: Invest Ophthalmol Vis Sci. 2022 Oct 3;63(11):1. doi: 10.1167/iovs.63.11.1 (PMC9547357; doi:10.1167/iovs.63.11.1)
Supplement: Supplement 2 [file iovs-63-11-1_s002.pdf]

Supplementary Materials

Investigative Ophthalmology & Visual Science
2022

COOPERATION OF ANGIOPOIETIN-2 AND ANGIOPOIETIN-4 IN SCHLEMM'S CANAL MAINTENANCE

Emmi Kapiainen,[1–3] Harri Elamaa,[1–3] Ilkka Miinalainen,[2,3] Valerio Izzi,[1,2,4,5] and Lauri Eklund[1–3]

[1]Oulu Center for Cell–Matrix Research, University of Oulu, Oulu, Finland
[2]Faculty of Biochemistry and Molecular Medicine, University of Oulu, Oulu, Finland
[3]Biocenter Oulu, University of Oulu, Oulu, Finland
[4]Faculty of Medicine, University of Oulu, Oulu, Finland
[5]Foundation for the Finnish Cancer Institute, Helsinki, Finland

Supplementary Materials and Methods
Supplementary Figures S1–S9
Supplementary Tables S1–S2
Supplementary Video S1 Legend

SUPPLEMENTARY MATERIALS AND METHODS

Immunofluorescence stainings

The eyes were fixed in 4% PFA for 30 min at room temperature (RT; or overnight at +4 °C) and stored in 1×PBS at +4 °C. For SC whole mount staining, corneal limbus region containing SC was dissected out either as whole corneal preparation or as two or three stripes, and the iris was carefully removed. 5% donkey serum–0.3% Triton X-100 in 1×PBS was used as the incubation solution in all staining steps. The samples were blocked for one hour at RT, incubated overnight with the primary antibodies at +4 °C, washed by changing the solution at least four times during two-three hours at RT, incubated overnight with secondary antibodies at +4 °C, washed, and flat-mounted with Immu-Mount (Thermo Fisher Scientific, Waltham, MA). The stripes were flat-mounted side by side in a vertical position the cornea always to the right to facilitate optimal imaging of the whole SC. Alternatively, whole SC regions from *Angpt4*$^{+/Cre}$; *Rosa26*$^{+/mTmG}$ reporter mice were also directly flat-mounted without any staining. Retina whole mounts were stained similarly as whole SC.

For sectioned tissue images of *Angpt4*$^{+/Cre}$; *Rosa26*$^{+/mTmG}$ mice, whole eyes were fixed with 95% ethanol overnight and processed for paraffin sectioning and dehydration as described elsewhere.[1] For cryosection staining of WT and *Angpt2*$^{-/-}$ mice, eyes were freshly frozen into Tissue-Tek OCT compound (Sakura, Alphen aan den Rijn, The Netherlands), cut to 10 µm sections and stored at -80 °C. Upon staining, sections were fixed with 4% PFA for 3 min, blocked with 5% donkey serum–0.3% Triton X-100 in 1×PBS for 30 min and incubated overnight with primary antibodies at +4 °C in a humified chamber followed by incubation with secondary antibodies for 2 h at RT and mounting with Immu-Mount.

The primary antibodies used were anti-CD31 (rat polyclonal, 550274, BD Pharmingen [Fair Lawn, NJ], RRID:AB_393571), anti-Lyve-1 (rabbit polyclonal, 11-034, AngioBio [San Diego, CA], RRID:AB_2813732), anti-Prox1 (goat polyclonal, AF2727, R&D Systems [Minneapolis, MN], RRID:AB_2170716), anti-Tie2 (goat polyclonal, AF762, R&D Systems, RRID:AB_2203220), anti-phospho-Tie2 (rabbit polyclonal, AF2720, R&D Systems, RRID:AB_442172), anti-Angpt2 (human monoclonal; a kind gift from Professor Gou Young Koh)[2,3] and anti-Angpt2 (sheep polyclonal, AF7186, R&D Systems, RRID:AB_10973828). Secondary antibodies used were Alexa Fluor 488–conjugated donkey anti-human (709-545-149, RRID:AB_2340566) and anti-goat (705-545-147, RRID:AB_2336933), Cy3-conjugated donkey anti-rabbit (711-165-152, RRID:AB_2307443), anti-goat (705-165-147, RRID:AB_2307351), and anti-sheep (713-165-147, RRID:AB_2315778), and Alexa Fluor 647–conjugated donkey anti-rat (712-605-153, RRID:AB_2340694) or anti-goat (705-

605-147, RRID:AB_2340437), all from Jackson ImmunoResearch (West Grove, PA). DAPI (4′,6-diamidino-2-phenylindole; D9542, Sigma Aldrich) was used to stain nuclei. All antibodies were diluted 1:300.

Confocal microscopy and morphometrical analyses

The fluorescently labeled samples were imaged with Zeiss LSM780 confocal microscope (Zeiss, Oberkochen, Germany) using Plan Apochromat 10×/0.45 air, 20×/0.8 air, or 40×/1.4 oil objectives. Excitation wavelengths were 405 nm for DAPI, 488 nm for Alexa Fluor 488, 561 nm for Cy3 or tdTomato, and 633 nm for Alexa Fluor 647. Emission was collected between 415–490 nm for DAPI, 490–543 nm for Alexa Fluor 488, 569–621 nm for Cy3 or 569–630 nm for tdTomato, and 638–755 nm for Alexa Fluor 647. Pixel size was set to 0.83 µm × 0.83 µm with 10×, 0.42 µm × 0.42 µm with 20×, and 0.21 µm × 0.21 µm with 40×, and the pinhole size was set to 1 AU. Z-stacks were taken at approximately 5 µm intervals for 10× and 20× images (morphometrical analyses) and with approximately 2 µm intervals for 40× images (Angpt expressions), and the images were processed to maximum intensity projections with Zen 2.3 lite software (Zeiss). 20× 1×2 tile images were taken carefully covering the whole SC (or as much as possible) for $CD31^+$ SC area, Prox1 expression, and pTie2/Tie2 expression measurements, while 10× larger tile images were taken from CD31 and Lyve1 stainings to image the whole corneolimbal area. $CD31^+$ SC area was measured from the 20× images using the Zen software. SC area was divided by the whole analyzed image area as a control, and the measurements from all images were averaged to obtain a single value for each eye. $Prox1^+$ nuclei in the SC were analyzed from the 20× images using Fiji (ImageJ) software. CD31 staining was first used to determine SC area. Uneven local background fluorescence was subtracted from Prox1 staining by subtracting the duplicated median filtered image from the original image (corrected image = image – median filtered image, radius 50). CD31 staining masked region of interest (ROI) was then thresholded with constant pixel values (17–255, set to include all but the very faintest positive nuclei), and the binarized image was median filtered with a radius of 2 pixels to remove remaining noise. Analyze particles–function was used to create ROI maps of the nuclei, and these ROI maps were then used to measure the number and area of $Prox1^+$ nuclei from the background-subtracted, non-binarized image. Finally, the number of $Prox1^+$ nuclei per mm^2 of $CD31^+$ SC area and the percentage of total $Prox1^+$ area from the $CD31^+$ SC area per eye were calculated. Tie2 and pTie2 intensity mean values were measured from the 20× images with the Zen software using CD31 staining to determine SC area; the background intensity values measured from non-SC areas of the images were subtracted

from the values, and after finding no differences in total Tie2 levels, pTie2/Tie2 ratio was reported. $Lyve1^+$ corneal limbus LV area was quantified from the 10× images using the Zen software. LV area was normalized with SC length measured from 10× images using Fiji; conjunctival LVs and $Lyve1^+$ limbal macrophages[4,5] were excluded from LV area analyses. Circulating limbal arteries and perilimbal veins which circulate the limbal area in a parallel manner,[6] collecting vein branches (including both collector channels with direct connections to the SC and perilimbal venous plexus branches at the sites of major vein drainage towards episcleral veins),[6] corneal arcades (loops of the limbal capillary plexus on the corneal side),[6] and SC narrowing points were calculated from the 10× images. SC narrowing points were determined as distinctive narrowings/convolutions/twists in the SC, and a new measurement point was assigned after every 100 µm if the SC continued as narrow.

scRNAseq data analyses

Previously published[7] scRNAseq data (data accessible at NCBI GEO database,[8] accession number GSE146188) were downloaded from the Broad Institute Single Cell Portal (http://singlecell.broadinstitute.org/) and reanalyzed in R using the Seurat package[9] following standard procedures as suggested by the package authors. The reanalyzed Seurat3 object is available upon request.

X-gal staining

Eyes from *Angpt4*^{LacZ} and control mice were fixed in 4% PFA for 10 min on ice, washed for 1 h with washing buffer (0.02% NP-40, 0.01% Sodium Deoxycholate, 2 mmol/L $MgCl_2$, 5 mmol/L EGTA in 0.1 mol/L phosphate buffer, pH 7.4), and incubated overnight in 1 mg/mL X-gal/10 mmol/L potassium ferro- and ferricyanide at RT. After 1 h washing, eyes were postfixed in 4% PFA for 30 min at RT, embedded in OCT compound, frozen, cut to 10 µm sections and immediately mounted with Immu-Mount. Sections were imaged with Zeiss Axio Imager motorized brightfield microscope.

Quantitative PCR

RNA was isolated from tissues using Fibrous Tissue Mini Kit (Qiagen, Germantown, MD). 1 µg (anterior eye) or 3 µg (kidney, lung) of total RNA was transcribed to cDNA, and qPCR was performed

as described previously[10] using *β-actin* or *Gapdh* as reference genes. Primers are reported in the Supplementary Table S1.

Transmission electron microscopy

TEM sample processing and imaging was performed as previously described.[10] The number of giant vacuoles per 100 µm of SC inner wall endothelium was measured with Fiji. Eyes for TEM analyses were always collected first after sacrificing a mouse to prevent inconsistent loss of vacuoles. A small hole was cut to the middle of the cornea after fixation to facilitate the access of the reagents during TEM sample processing.

Retinal ganglion cell and nerve fiber layer analysis

Eyes were fixed in Davidson's fixative (2% formalin, 30% ethanol, 10% acetic acid in distilled water) for 1–2 days at +4 °C and processed for paraffin sectioning (5 µm sections from the center of the eye containing the optic nerve head) and standard hematoxylin and eosin (H&E) staining. Sections were scanned with Hamamatsu NanoZoomer S60 slide scanner with a 40× objective. The retinal nerve fiber layer (RNFL) thickness was measured with NDP.view 2 software starting at approximately 300 µm away from the optic nerve head for 500 µm length of both left and right side of the retina from 10 points on each side with regular intervals. These 20 measurements were then averaged to obtain a single value for each eye. Retinal ganglion cell (RGC) nuclei were calculated from the same area from both left and right side of the retina and averaged to obtain a single value for each eye.

SUPPLEMENTARY REFERENCES

1. Nakagawa A, von Alt K, Lillemoe KD, Fernández-Del Castillo C, Warshaw AL, Liss AS. A method for fixing and paraffin embedding tissue to retain the natural fluorescence of reporter proteins. *Biotechniques*. 2015;59(3):153-155. doi:10.2144/000114328
2. Park DY, Lee J, Kim J, et al. Plastic roles of pericytes in the blood-retinal barrier. *Nature Communications*. 2017;8:15296. doi:10.1038/ncomms15296

3. Kapiainen E, Kihlstrom MK, Pietilä R, et al. The Amino-Terminal Oligomerization Domain of Angiopoietin-2 Affects Vascular Remodeling, Mammary Gland Tumor Growth, and Lung Metastasis in Mice. *Cancer Research*. 2021;81(1):129–143. doi:10.1158/0008-5472.CAN-19-1904

4. Wu Y, Seong YJ, Li K, et al. Organogenesis and distribution of the ocular lymphatic vessels in the anterior eye. *JCI Insight*. 2020;5(13):e135121. doi:10.1172/jci.insight.135121

5. Xu H, Chen M, Reid DM, Forrester J v. LYVE-1-positive macrophages are present in normal murine eyes. *Investigative Ophthalmology and Visual Science*. 2007;48(5):2162–2171. doi:10.1167/iovs.06-0783

6. van der Merwe EL, Kidson SH. The three-dimensional organisation of the post-trabecular aqueous outflow pathway and limbal vasculature in the mouse. *Experimental Eye Research*. 2014;125:226–235. doi:10.1016/j.exer.2014.06.011

7. van Zyl T, Yan W, McAdams A, et al. Cell atlas of aqueous humor outflow pathways in eyes of humans and four model species provides insight into glaucoma pathogenesis. *Proc Natl Acad Sci U S A*. 2020;117(19):10339–10349. doi:10.1073/pnas.2001250117

8. Edgar R, Domrachev M, Lash AE. Gene Expression Omnibus: NCBI gene expression and hybridization array data repository. *Nucleic Acids Research*. 2002;30(1):207–210. doi:10.1093/nar/30.1.207

9. Hao Y, Hao S, Andersen-Nissen E, et al. Integrated analysis of multimodal single-cell data. *Cell*. 2021;184(13):3573–3587.e29. doi:10.1016/j.cell.2021.04.048

10. Elamaa H, Kihlström M, Kapiainen E, et al. Angiopoietin-4-dependent venous maturation and fluid drainage in the peripheral retina. *Elife*. 2018;7:e37776. doi:10.7554/eLife.37776

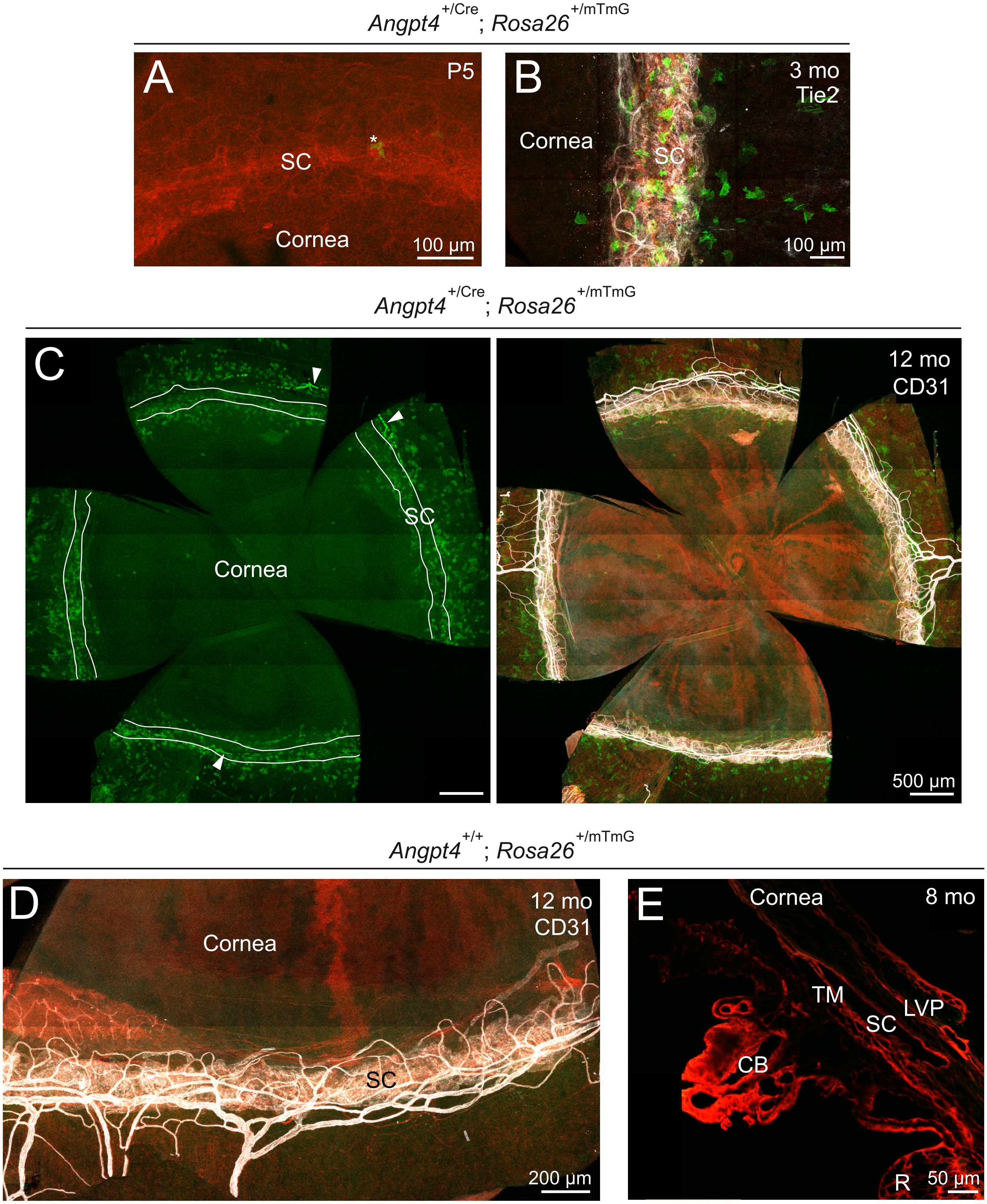

Supplementary Figure S1. Supplemental images of Angpt4 expression in the iridocorneal angle, and negative controls. (**A**) At P5, *Angpt4*$^{+/Cre}$; *Rosa26*$^{+/mTmG}$ mice show only marginal *Angpt4*^{Cre} promoter–induced GFP expression in very few SC surrounding cells (asterisk). (**B**) GFP^{+} cells in 3 month old *Angpt4*$^{+/Cre}$; *Rosa26*$^{+/mTmG}$ mouse locate close to SC (stained with Tie2). (**C**) Whole corneal flat mount of an adult *Angpt4*$^{+/Cre}$; *Rosa26*$^{+/mTmG}$ mouse. GFP^{+} cells evenly encircle the whole SC around the eye. Arrowheads point to the limbal arteries in which *Angpt4*^{Cre} is expressed in the smooth muscle cells. CD31 staining (white) visualizes the SC (marked with white lines on the left) and surrounding blood and lymphatic vasculature. (**D–E**) *Angpt4*$^{+/+}$; *Rosa26*$^{+/mTmG}$ mice are devoid of GFP^{+} cells, as expected without Cre expression. SC is stained with CD31 (white). CB, ciliary body; LVP, limbal vascular plexus; R, retina; SC, Schlemm's canal; TM, trabecular meshwork.

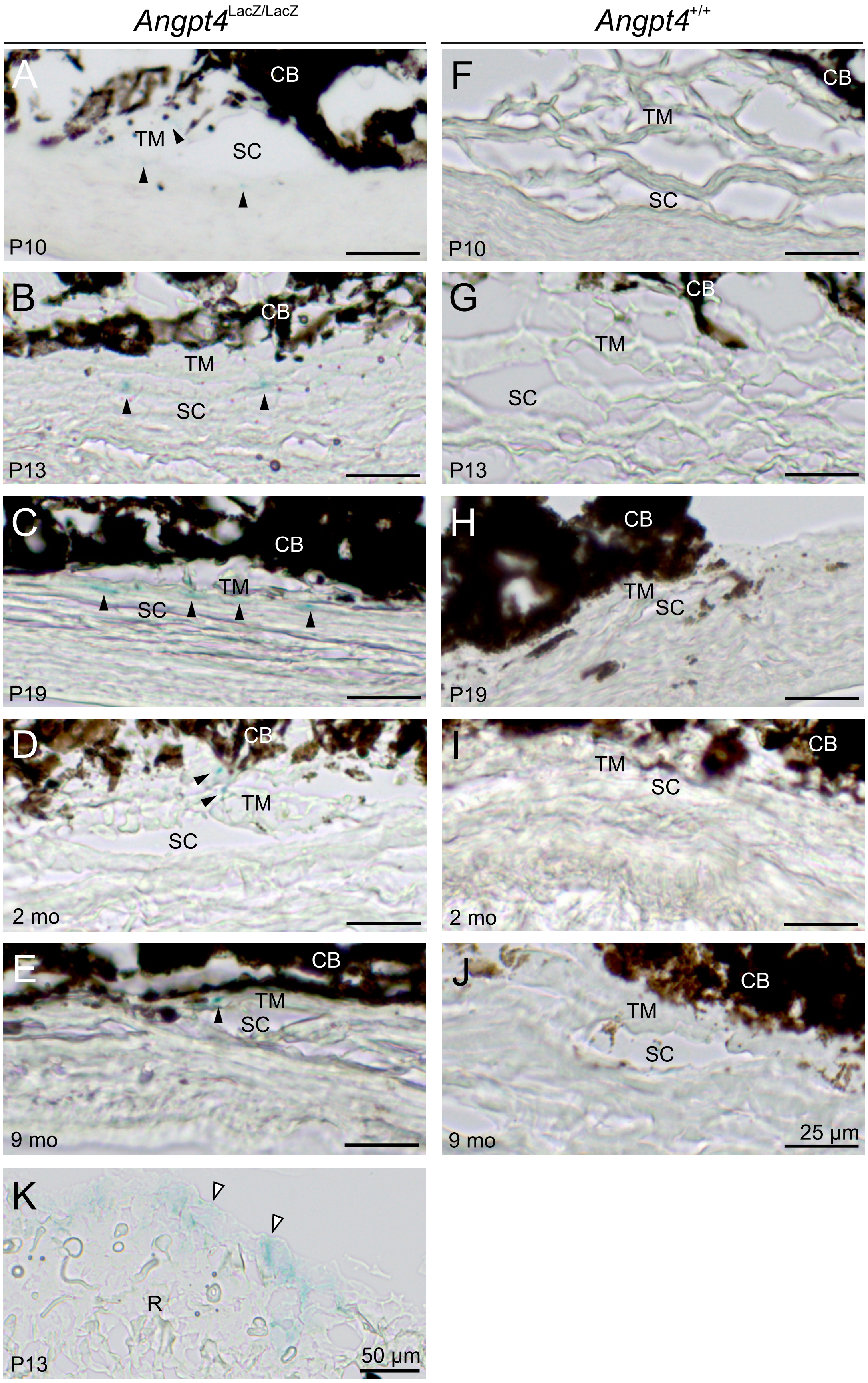

Supplementary Figure S2. ***Angpt4*****^{LacZ} reporter mice show *Angpt4* expression in the trabecular meshwork.** On the left, *Angpt4*^{LacZ} mice express β-galactosidase enzyme under *Angpt4* promoter (**A**–**E**). X-gal staining of *Angpt4*$^{LacZ/LacZ}$ mice visualizing normal *Angpt4* expression sites reveals only a few faintly positive cells (blue precipitate, arrowheads) in the trabecular meshwork (TM) at P10 (**A**), and a few more intensely stained cells at P13 (**B**), P19 (**C**), 2 months (**D**), and 9 months (**E**). On the right, correspondingly aged *Angpt4*$^{+/+}$ mice were negative controls of staining (**F**–**J**). (**K**) Retinal (R) astrocytes (white arrowheads) were internal positive controls of staining. CB, ciliary body.

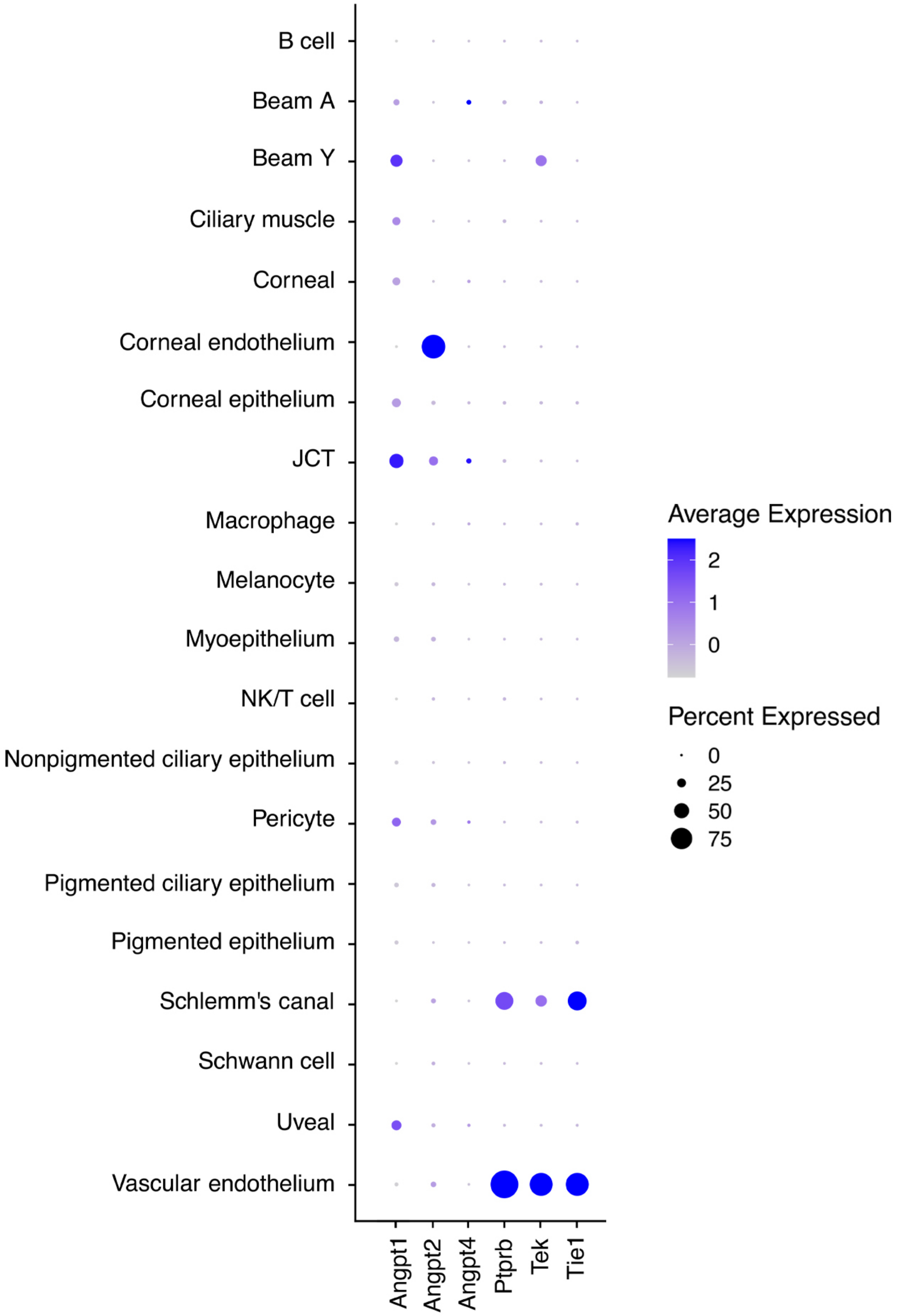

Supplementary Figure S3. scRNAseq analysis of the expression of Angpts, Tie receptors, and VE-PTP in the mouse iridocorneal angle. Dots represent the expression profiles for the given genes across the different cell populations (cell clustering follows the original data by van Zyl et al., 2020). Dot diameter is proportional to the percentage of cells expressing the given genes at any level, while the color intensity is proportional to the average gene expression in the population. Note that *Tek* is the gene name of Tie2 and *Ptprb* of VE-PTP. Beam A, beam Y, and JCT (juxtacanalicular tissue) represent the trabecular meshwork cells.

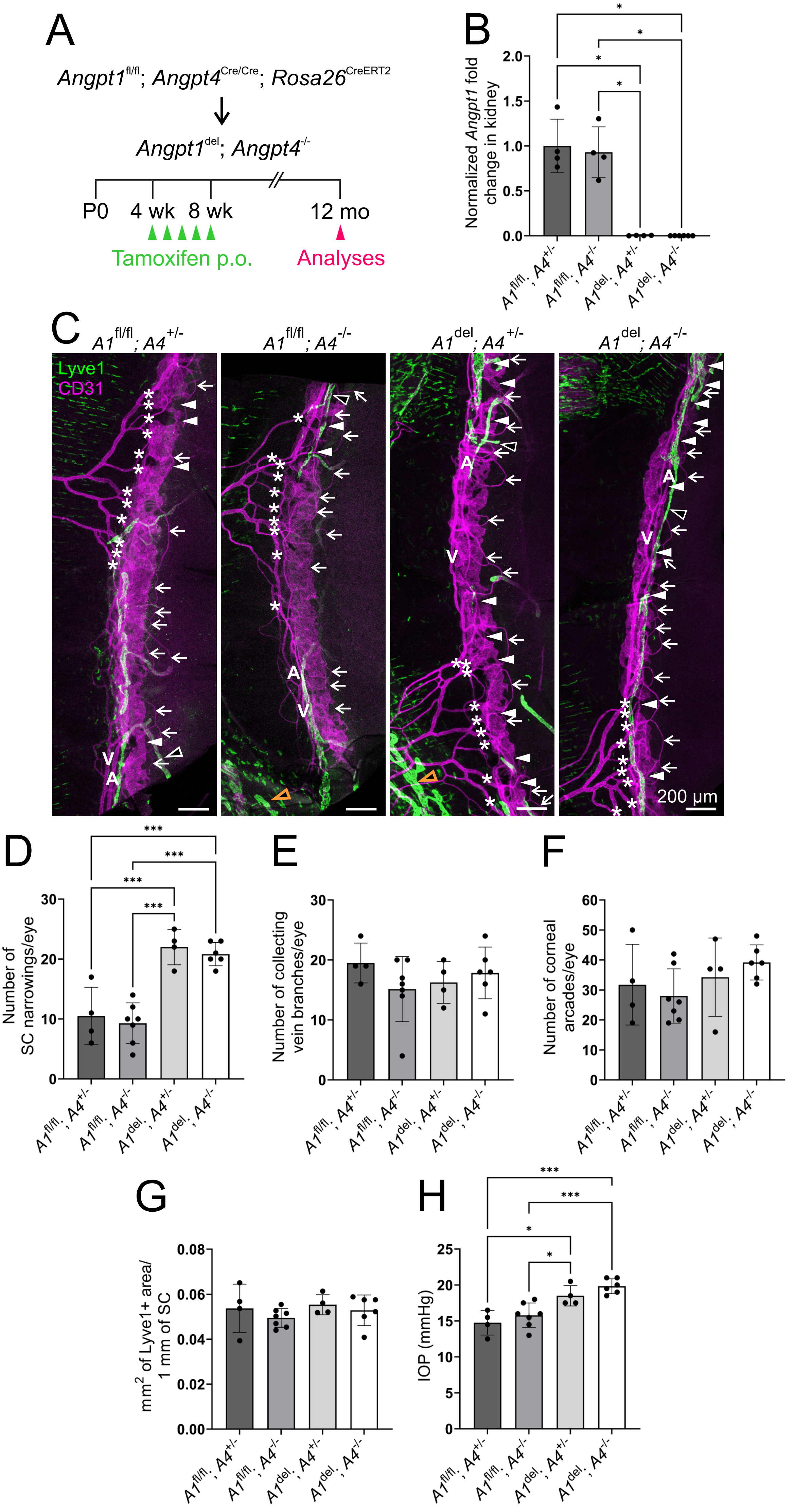
A
Angpt1fl/fl; Angpt4Cre/Cre; Rosa26CreERT2
Angpt1del; Angpt4-/-
P0
4 wk
8 wk
12 mo
Tamoxifen p.o.
Analyses
B
Normalized Angpt1 fold change in kidney
C
Lyve1
CD31
200 µm
D
Number of SC narrowings/eye
E
Number of collecting vein branches/eye
F
Number of corneal arcades/eye
G
mm2 of Lyve1+ area/ 1 mm of SC
H
IOP (mmHg)
A1fl/fl, A4+/-
A1fl/fl, A4-/-
A1del, A4+/-
A1del, A4-/-

Supplementary Figure S4. SC morphology analyses in *Angpt1*^{del}; *Angpt4*$^{-/-}$ mice. (**A**) Generation and analysis of *Angpt1*; *Angpt4* double deficient mice. Ubiquitous *Angpt1* deletion in *the Angpt1*$^{fl/fl}$*; Angpt4*$^{Cre/Cre}$*; Rosa26*^{CreERT2} mice (later abbreviated as *A1*^{del}; *A4*$^{-/-}$ mice) was induced by weekly oral administration of tamoxifen at 4–8 weeks of age. Analyses were carried out when the mice had aged for 1 year. fl, floxed. (**B**) *Angpt1* deletion efficiency was confirmed from the kidney. qPCR analysis shows absence of *Angpt1* mRNA in the *A1*^{del} mice. (**C**) Representative images of corneal limbus area. CD31 (magenta) strongly labels the SC and limbal blood vasculature, and more faintly the lymphatic vessels (LVs). Lyve-1 (green) distinctly marks the LVs (open arrowheads) and also limbal macrophages. A, circular limbal artery; V, perilimbal vein; these vessels were found in 4/4 *A1*$^{fl/fl}$; *A4*$^{+/-}$, 7/7 *A1*$^{fl/fl}$; *A4*$^{-/-}$, 4/4 *A1*^{del}; *A4*$^{+/-}$ and 6/6 *A1*^{del}; *A4*$^{-/-}$ mice analyzed. Arrowheads mark SC narrowing points quantified in (**D**), asterisks mark collector vein branches quantified in (**E**), arrows mark corneal arcades of the limbal capillary plexus quantified in (**F**), and Lyve1^{+} corneolimbal LVs were quantified in (**G**). Occasional conjunctival lymphatics are marked with orange-lined arrowheads in (**C**) and were not included in analysis in (**G**). (**H**) IOP measurements. n = 4/7/4/6 mice/genotype. *P<0.05, and ***P<0.001 in one-way ANOVA followed by Tukey post hoc test in **D**–**H**, or in Welch's ANOVA followed by Dunnett's T3 post hoc test in **B**.

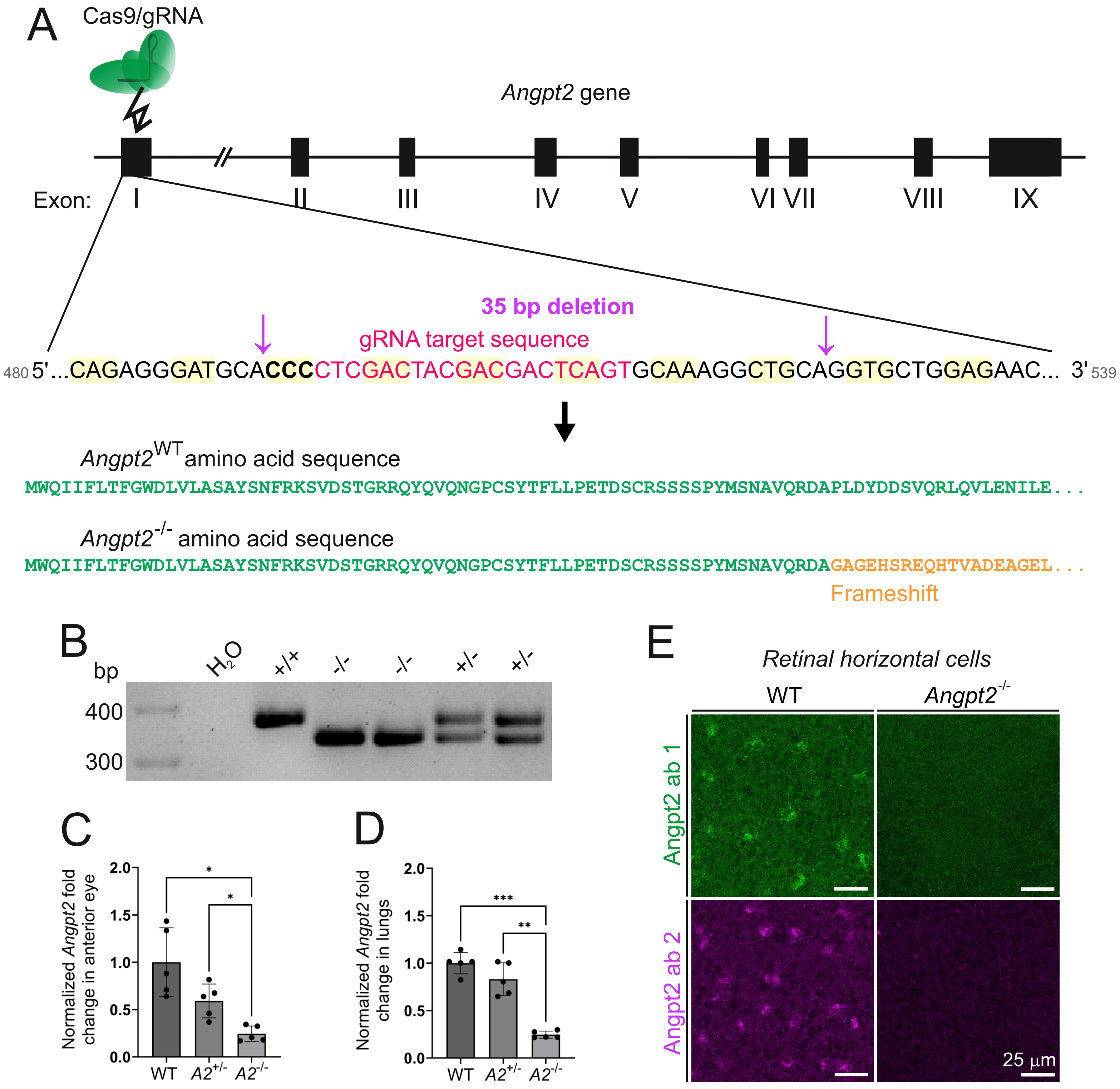

Supplementary Figure S5. Generation and characterization of *Angpt2* deletion mouse line. (**A**) CRISPR/Cas9 method was used to generate a frameshift into *Angpt2* exon 1. Deleted 35 bp sequence is shown with magenta arrows. GuideRNA (gRNA) target sequence, pink; protospacer adjacent motif, bolded; codons, yellow background. WT amino acid sequence is shown in green letters and the hypothetical amino acid sequence after the frameshift in orange letters. (**B**) PCR with primers flanking the deletion site shows a 373 bp product from WT (+/+) and 338 bp product from deleted *Angpt2* allele (-/-). (**C–D**) qPCR analysis of *Angpt2* mRNA levels in the anterior eye (**C**) and lungs (**D**) of WT, *Angpt2*$^{+/-}$ (*A2*$^{+/-}$) and *Angpt2*$^{-/-}$ (*A2*$^{-/-}$) mice normalized to WT. *n* = 5 mice/genotype, **P*<0.05, ***P*<0.01, ****P*<0.001 in Welch's ANOVA followed by Dunnett's T3 post hoc test. (**E**) Immunofluorescent stainings of mouse retinal horizontal cells with two distinct Angpt2 antibodies (ab 1, green, a gift from G.Y. Koh; ab 2, magenta, cat. no. AF7186, R&D Systems) show absence of Angpt2 protein in *Angpt2*$^{-/-}$ mice.

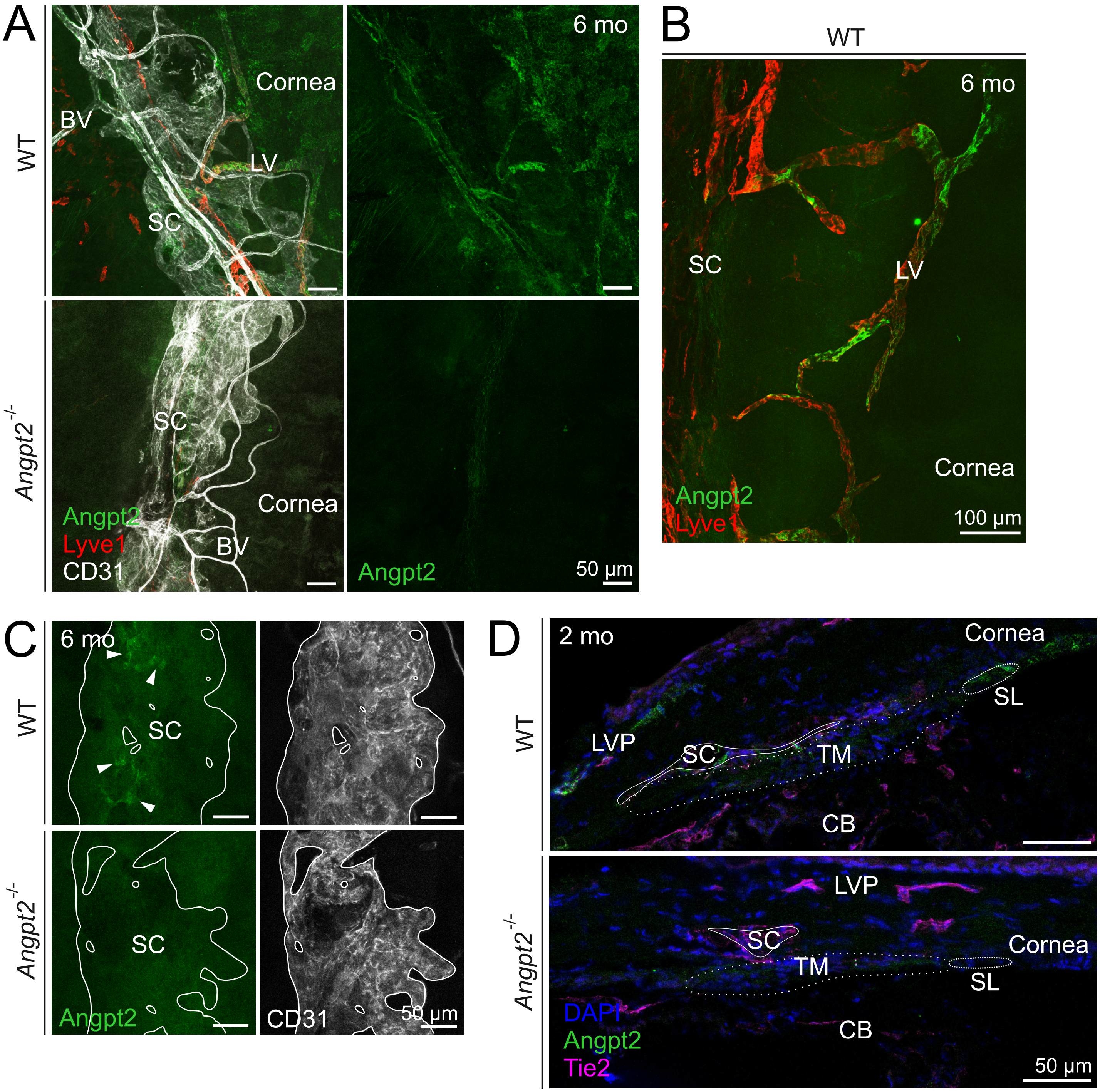

Supplementary Figure S6. Angpt2 staining localizes to the corneal limbus area. Antibody staining shows Angpt2 (green) localization in the flat-mounted cornea, in the blood vessels (BV) surrounding SC (CD31, white), in Lyve1^{+} (red) corneal limbus lymphatic vessels (LV) (**A**–**B**), and in occasional SC endothelial cells (arrowheads) (**C**) in 6 months (mo) old mice. SC is separated by solid white lines in (**C**). (**D**) In cryosectioned tissue from 2 mo old mice, Angpt2 is localized to limbal vascular plexus (LVP), corneal and SC endothelium, and to Schwalbe line cells (SL), a separate non-filtering insert region of trabecular meshwork (TM). On the contrary, only relatively weak Angpt2 staining is seen in the filtrating TM. TM is separated by dashed lines, SL by dense dashed lines, and SC by solid lines. Tie2 (magenta) marks endothelial cells and DAPI (blue) nucleus.

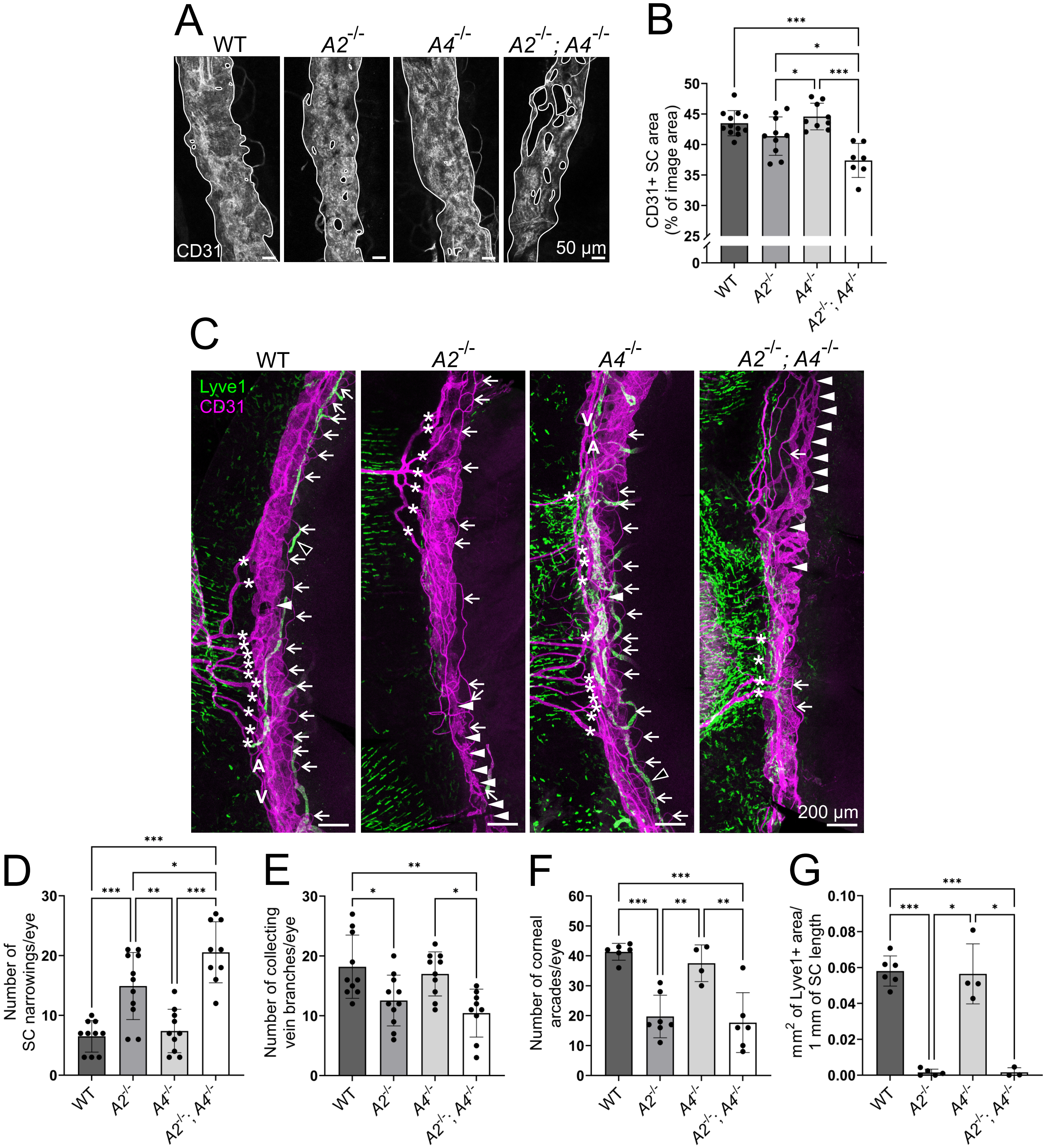

Supplementary Figure S7. Schlemm's canal morphology in 12-weeks-old mice depleted of *Angpt2* and *Angpt4*. (**A**) Representative close-up images of CD31 antibody staining (white) of whole mount SC. SC area was quantified in (**B**). *n* = 12/10/9/7 mice/genotype. (**C**) Representative images of corneal limbus area, CD31 (magenta) labeling the SC and limbal blood vasculature, and Lyve1 (green) marking lymphatic vessels (open arrowheads). A, circular limbal artery; V, perilimbal vein; these vessels were found in 6/6 WT, in 2/7 (A) and 1/7 (V) *Angpt2*$^{-/-}$, in 4/4 *Angpt4*$^{-/-}$, and in 1/6 (A) and 0/6 (V) *Angpt2*$^{-/-}$; *Angpt4*$^{-/-}$ mice analyzed. Arrowheads mark SC narrowing points quantified in (**D**), asterisks mark collecting vein branching points quantified in (**E**), arrows mark corneal arcades of the limbal capillary plexus quantified in (**F**), and Lyve1^{+} corneolimbal lymphatics were quantified in (**G**). *n* = 10/11/10/9 mice/genotype in **D** and **E**, *n* = 6/7/4/6 mice/genotype in **F**, and *n* = 6/5/4/3 mice/genotype in **G**. **P*<0.05, ***P*<0.01, ****P*<0.001 in one-way ANOVA followed by Tukey post hoc test in **B** and **D**–**F**, or in Welch's ANOVA followed by Dunnett's T3 post hoc test in **G**.

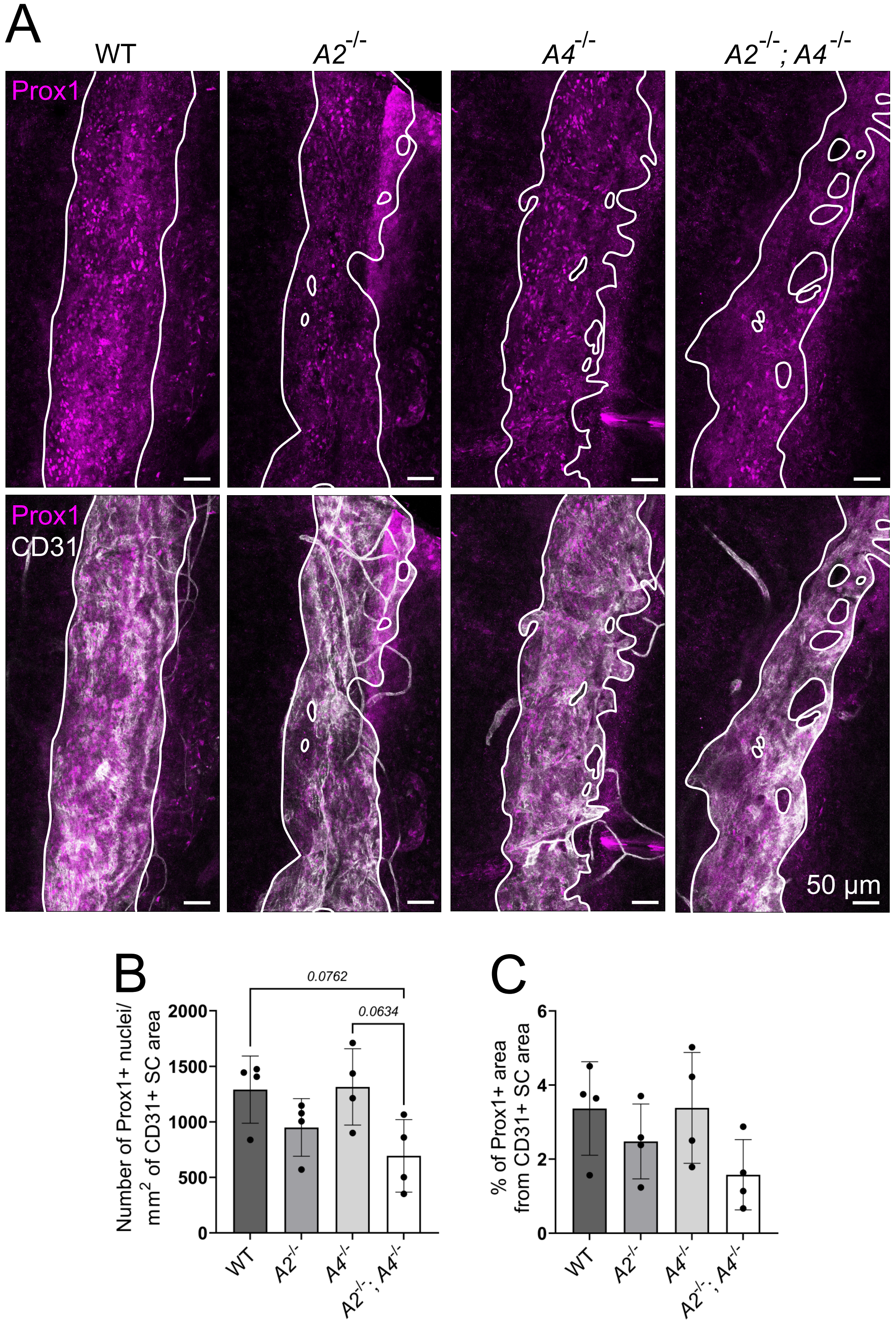

Supplementary Figure S8. Prox1 expression in the Schlemm's canal of 12-weeks-old *Angpt2*- and *Angpt4*-deficient mice. (**A**) Representative close-up images of Prox1 (magenta) and CD31 (white) antibody staining of whole mount SC. Number and area of $Prox1^+$ nuclei in relation to $CD31^+$ SC area (defined with white lines) was quantified in (**B–C**). $n = 4$ mice/genotype. Statistical significances were tested with one-way ANOVA followed by Tukey post hoc test.

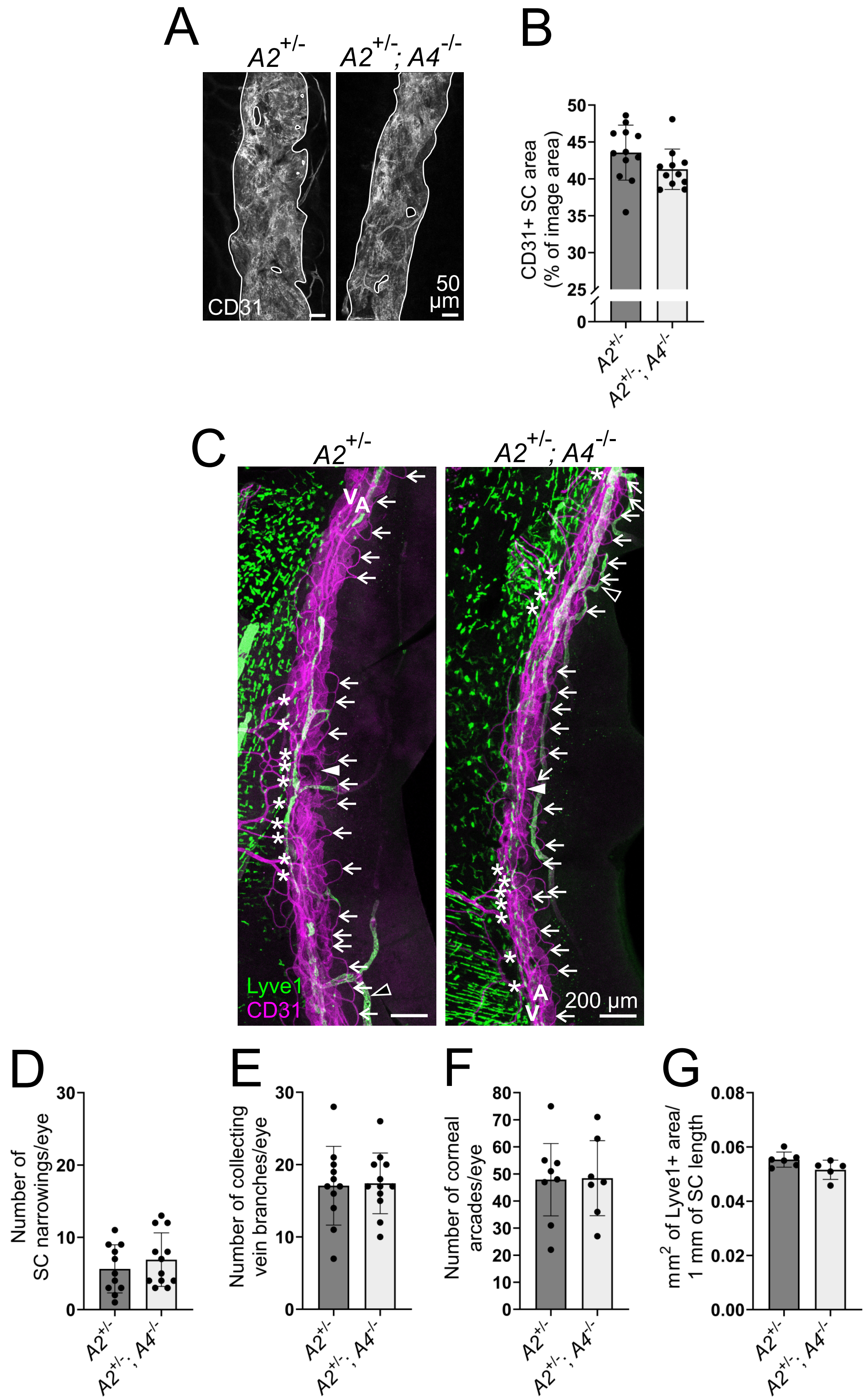

Supplementary Figure S9. Schlemm's canal morphology in 12-weeks-old *Angpt2*$^{+/-}$ and *Angpt2*$^{+/-}$; *Angpt4*$^{-/-}$ mice. (**A**) Representative close-up images of CD31 antibody staining (white) of whole mount SC. SC area was quantified in (**B**). (**C**) Representative images of corneal limbus area, CD31 (magenta) labeling the SC and limbal blood vasculature, and Lyve1 (green) marking lymphatic vessels (open arrowheads). A, circular limbal artery; V, perilimbal vein; these vessels were found in 8/8 *Angpt2*$^{+/-}$ and 7/7 *Angpt2*$^{+/-}$; *Angpt4*$^{-/-}$ mice analyzed. Arrowheads mark SC narrowing points quantified in (**D**), asterisks mark collecting vein branches quantified in (**E**), arrows mark corneal arcades of the limbal capillary plexus quantified in (**F**), and Lyve1^{+} corneolimbal lymphatics were quantified in (**G**). n = 11–12 mice/genotype in **B**, **D** and **E**, n = 7–8 mice/genotype in **F**, and n = 5–6 mice/genotype in **G**. Unpaired Student's two-tailed t-test was used to test statistical significances.

Supplementary Table S1. Primers used for mouse line genotyping and mouse tissue qPCR.

Primer	Sequence	Description
Angpt1 forward	GGGGGAGGTTGGACAGTAA	*Angpt1*, qPCR
Angpt1 reverse	CATCAGCTCAATCCTCAGC	*Angpt1*, qPCR
Angpt1 fl forward	CATCTGGAGCATGTGATGGA	floxed *Angpt1*, genotyping
Angpt1 fl reverse	GTGCGCGCTTACTTTTTCA	floxed *Angpt1*, genotyping
Angpt1 del forward	TCCTGGTCTTTGCACTTGACTG	*Angpt1* deletion, genotyping
Angpt1 del reverse	GTGCGCGCTTACTTTTTCA	*Angpt1* deletion, genotyping
Angpt2 forward	GACAGCAGCTGAGAGCTCAGG	*Angpt2*, genotyping
Angpt2 reverse	CCAGCCCACCTTCCTACCTTC	*Angpt2*, genotyping
Angpt2 forward	TTAGCACAAAGGATTCGGACAAT	*Angpt2*, qPCR
Angpt2 reverse	TTTTGTGGGTAGTACTGTCCATTCA	*Angpt2*, qPCR
Angpt4 forward	TCCTTAAAGACACCTAAGCCAGTG	*Angpt4*, qPCR
Angpt4 reverse	GGTCCTCTGGAAATTTACGCTTCC	*Angpt4*, qPCR
β-actin forward	TGTTACCAACTGGGACGACA	*β-actin*, qPCR
β-actin reverse	GGGGTGTTGAAGGTCTCAAA	*β-actin*, qPCR
CreERT2 forward	TGATTGGTCTCGTCTGGCGCT	*Rosa26*CreERT2, genotyping
CreERT2 reverse	TAGAGGGGCACCACGTTCTTGC	*Rosa26*CreERT2, genotyping
Gapdh forward	GGTGAAGGTCGGTGTGAACG	*Gapdh*, qPCR
Gapdh reverse	CTCGCTCCTGGAAGATGGTG	*Gapdh*, qPCR
Tie2 forward	ATGTGGAAGTCGAGAGGCGAT	*Tie2*, qPCR
Tie2 reverse	CGAATAGCCATCCACTATTGTCC	*Tie2*, qPCR

Supplementary Table S2. Proportions of cells expressing *Angpt1*, *Angpt4*, or both in such cell types that do express *Angpt4* according to the reanalyzed scRNAseq data (van Zyl et al., 2020). Only the *Angpt4*-expressing cell types are included. Note that beam A and JCT (juxtacanalicular tissue) are specific types of trabecular meshwork cells.

**Cell population based on scRNAseq expression profile**	**% of *Angpt1*+ cells out of whole cell population indicated**	**% of *Angpt4*+ cells out of whole cell population indicated**	**% of double+ cells out of whole cell population indicated**	**% of *Angpt1*+ cells also expressing *Angpt4***	**% of *Angpt4*+ cells also expressing *Angpt1***
Beam A	12.50	7.50	0	0	0
Corneal	19.05	2.38	0	0	0
Corneal epithelium	22.69	1.68	1.26	5.56	75.00
JCT	42.70	8.99	4.49	10.52	49.94
Macrophage	0.25	1.26	0	0	0
Pericyte	22.62	2.38	0	0	0
Uveal	26.59	2.08	1.46	5.49	70.19

SUPPLEMENTARY VIDEO S1 LEGEND

Animation of consecutive z-stacks taken with a confocal microscope across a corneal limbus sample, which has been mounted between two glass slides. In the *Angpt4*$^{+/Cre}$; *Rosa26*$^{mTmG/+}$ mice, cells that express of have expressed *Angpt4*^{Cre} are permanently GFP-labeled (green). CD31 antibody staining (magenta) labels both the first appearing limbal vascular plexus and the later emerging Schlemm's canal (SC) endothelium (image plane moving from the outer eye towards the inner eye). The GFP^{+} cells do not overlap with the SC endothelium since there is minimal amount of white signal which would indicate merging of green GFP and magenta CD31 signals; however, the GFP^{+} cells locate closely to the SC, and also on the stroma nearer the outer eye.
